# Integrated Physiological, Biochemical, and Molecular Analysis Identifies Important Traits and Mechanisms Associated with Differential Response of Rice Genotypes to Elevated Temperature

**DOI:** 10.3389/fpls.2015.01044

**Published:** 2015-11-27

**Authors:** Boghireddy Sailaja, Desiraju Subrahmanyam, Sarla Neelamraju, Turaga Vishnukiran, Yadavalli Venkateswara Rao, Pujarula Vijayalakshmi, Sitapati R. Voleti, Vijai P. Bhadana, Satendra K. Mangrauthia

**Affiliations:** ^1^Biotechnology, Indian Institute of Rice ResearchHyderabad, India; ^2^Plant Physiology, Indian Institute of Rice ResearchHyderabad, India; ^3^National Professor Lab, Indian Institute of Rice ResearchHyderabad, India; ^4^Plant Breeding, Indian Institute of Rice ResearchHyderabad, India

**Keywords:** heat stress, *Oryza sativa*, Hsf, antioxidants, photosynthesis

## Abstract

In changing climatic conditions, heat stress caused by high temperature poses a serious threat to rice cultivation. A multiple organizational analysis at physiological, biochemical, and molecular levels is required to fully understand the impact of elevated temperature in rice. This study was aimed at deciphering the elevated temperature response in 11 popular and mega rice cultivars widely grown in India. Physiological and biochemical traits specifically membrane thermostability (MTS), antioxidants, and photosynthesis were studied at vegetative and reproductive phases, which were used to establish a correlation with grain yield under stress. Several useful traits in different genotypes were identified, which will be an important resource to develop high temperature-tolerant rice cultivars. Interestingly, Nagina22 emerged as the best performer in terms of yield as well as expression of physiological and biochemical traits at elevated temperature. It showed lesser relative injury, lesser reduction in chlorophyll content, increased super oxide dismutase, catalase and peroxidase activities, lesser reduction in net photosynthetic rate (*P*_*N*_), high transpiration rate (*E*), and other photosynthetic/fluorescence parameters contributing to least reduction in spikelet fertility and grain yield at elevated temperature. Furthermore, expression of 14 genes including heat shock transcription factors and heat shock proteins was analyzed in Nagina22 (tolerant) and Vandana (susceptible) at flowering phase, strengthening the fact that N22 performed better at molecular level also during elevated temperature. This study shows that elevated temperature response is complex and involves multiple biological processes that are needed to be characterized to address the challenges of extreme conditions of future climate.

## Introduction

Rice production and productivity are seriously affected by several biotic (diseases and insects) and abiotic (drought, extreme temperature, salinity, submergence, and heavy metals) stresses. In changing climatic conditions, these stresses have become more challenging and have already shown severe negative consequences in rice cultivation (Nguyen, 2002; Wassmann and Dobermann, 2007). Heat stress is one of the most serious issues in climate change, which affects all the phases of rice plant growth and metabolism (Prasad et al., 2006; Jagadish et al., 2008; Sailaja et al., 2014). Increase in daytime temperature to more than 34°C decreased rice yield up to 8% (Bahuguna et al., 2014; Shi et al., 2014). In 2003, about 5.18 million tons of paddy was lost due to heat wave with the temperature above 38°C for more than 20 days (Xia and Qi, 2004; Yang et al., 2004). The global mean temperature is rising every year and it is predicted that rise will be up to 3.7°C by 2100 (IPCC, 2013). These circumstances propel breeders to develop heat-tolerant rice cultivars that can sustain high temperature without yield penalty to a significant scale. A detailed analysis of biochemical and physiological processes contributing tolerance/susceptibility in rice is necessary to develop heat stress-tolerant rice cultivars (Krishnan et al., 2011). Several investigations have been carried out in rice to decipher the most sensitive phase and physiological processes affected by high temperature (Jagadish et al., 2010, 2011; Shi et al., 2014). Although both vegetative and reproductive phases are affected due to high temperature, the latter seems to be more crucial, thereby impacting the yield directly (Hall, 1992; Prasad et al., 2006; Jagadish et al., 2007, 2008, 2010; Shi et al., 2014).

Several studies have been conducted to identify rice genotypes tolerant to high temperature (Ishimaru et al., 2010; Jagadish et al., 2010; Prasanth et al., 2012; Ye et al., 2012); however, very few studies were aimed to study high temperature response in popular and mega rice cultivars (Ziska et al., 1996; Prasad et al., 2006; Shi et al., 2014). In addition, most of the earlier studies of heat stress treatment were based on sudden exposure of plants to a definite increased temperature for few hours or days, which causes a shock to plant cells. Indeed, these studies have provided substantial information of rice response to high temperature. However, such circumstances are unlikely to prevail in natural environment.

As the biological processes underlying rice responses to climate change are poorly understood, a comprehensive study comprising physiological, biochemical, and molecular analysis was performed, using popular rice cultivars exposed to elevated temperature. In this study, rice genotypes were grown at control and elevated temperatures right from seedling to maturity. Different physiological and biochemical traits, such as membrane thermostability (MTS), chlorophyll and carotenoid contents, antioxidant enzymes, and photosynthetic and fluorescence parameters, were measured at vegetative and reproductive phases. Yield attributes under control and elevated temperatures were utilized for correlation analysis with physiological traits to identify the most reliable traits for phenotyping or breeding of rice genotypes for elevated temperature tolerance. Furthermore, expression of 14 genes was analyzed in representative susceptible and tolerant rice cultivars.

## Materials and methods

The experiments were conducted to investigate the physiological and biochemical responses of selected popular rice cultivars (Table 1) at elevated temperature. Unlike other experiments where heat stress was applied by exposing plants to high temperature for short duration (1–2 h), this experiment was designed to study the response of genotypes growing at higher temperature, as 24-day-old seedlings were shifted to elevated temperature and maintained till harvesting of seeds. In order to simulate the elevated temperature treatment like natural environment, stress was imposed by shifting plants into a custom-made polyhouse that was built using metal frames and covered with transparent polythene sheets. Temperature inside and outside the polyhouse was recorded regularly (Supplementary Figure 1). Importantly, elevated temperature stress (inside polyhouse) was always proportional to the control (outside polyhouse) temperature. The plants were allowed to grow inside the polyhouse until physiological maturity. The mean of maximum and minimum temperature recorded from transplantation to flowering phase was 5.6 and 1.5°C higher, respectively, inside the polyhouse than outside. The mean maximum temperature from flowering to seed maturity period was 5.5°C higher inside the polyhouse.

**Table 1:** **List of rice (*Oryza sativa* subsp. indica) genotypes used in this study**.

**S. No.**	**Cultivar name (*Cv.*)**	**Parents**	**Year of release**
1	BPT5204	RP5 × Mahsuri	1986
2	IR64	IR2061-465-1-5-5 × IR657-33-2-1	1991
3	Jaya	TN1 × T141	1968
4	Krishna Hamsa	Fine Gora × Rasi	1997
5	MTU1010	Krishnaveni × IR64	2000
6	N22	Selection from Rajbhog	1978
7	Rasi	TN1 × CO29	1977
8	Sampada	Vijaya × C14-8	2008
9	Swarna	Vasistha × Mahsuri	1979
10	Vandana	C22 × Kalakeri	1992
11	Varadhan	Swarna × BR327-36	2008

**Figure 1:**
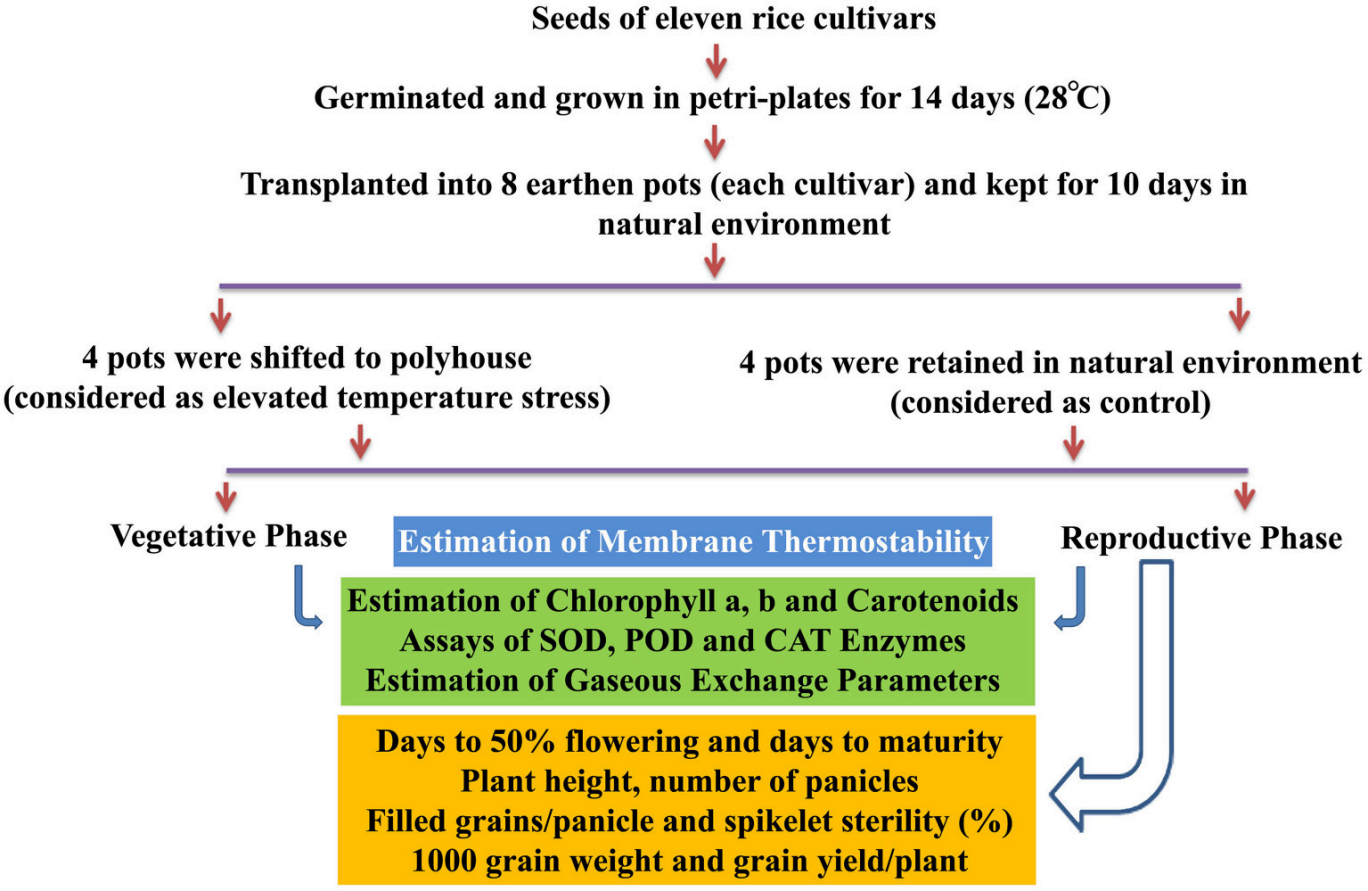
**Methodological framework of experiments conducted in this study**.

A methodological framework of experiments conducted in this study is shown in Figure 1. The experiment was carried out in Rabi season (January–May) of the years 2013 and 2014, which is considered as the best cropping season at Hyderabad, India, to study the heat stress experiments. The plants of 11 cultivars kept at control and elevated temperatures were used for physiological, biochemical, and yield studies at vegetative and reproductive phases. Fully matured leaf during vegetative stage and flag leaf after anthesis at reproductive stage were used for physiological and biochemical assays. The details of protocols and methods followed for estimation of MTS, chlorophyll, carotenoids, enzymes (SOD, CAT, and POD), gaseous exchange parameters, and yield attributes are given in Supplementary File 1.

### Statistical analysis

The data was analyzed by Analysis of Variance (ANOVA) using a statistical computer package Statistix Ver. 8.1. It was analyzed as per CRD (Completely Randomized Design). The differences between treatments and cultivars were estimated using HSD (Honest Significant Difference) test.

### Gene expression analysis

To study the gene expressions in susceptible and tolerant rice genotypes, seeds of N22 and Vandana cultivars were germinated in petri plates and transferred into earthen pots. One pot containing four plants of each cultivar was transferred to growth chamber for heat stress treatment. Heat stress (42°C) for 24 h was imposed during flowering initiation stage. Three biological replications were kept for this experiment.

Gene sequences were retrieved from NCBI (http://www.ncbi.nlm.nih.gov). Thirteen genes studied previously during heat stress experiment in rice seedlings (Sailaja et al., 2014) were used here for expression analysis. These were heat shock transcription factors (*OsHsfA2a, OsHsfA2e, OsHsfA7*), heat shock proteins (*HSP70* and *HSP81.1*), super oxide dismutase (*SOD*), sucrose-phosphate synthase 1 (*SPS*), cytochrome c oxidase assembly protein (*Cyt-C-Oxi*), squamosa promoter-binding-like protein 10 (*SPL*), cell wall integrity protein (*CWIP*), auxin response factor (*ARF*), nuclear transcription factor-Y (*NF-Y*) subunit A-3, and unknown protein similar to ferredoxin (*OsFd*). In addition to these genes, expression of a fertility restorer homolog gene (*FRH*, AK101861; forward primer 5′-TTACGCCACGCTGATTGAGG-3′ and reverse primer 3′-CCGCTCCGCATTACACAACC-5′) was also analyzed in this study.

Details of genes sources, primers, and methods followed for RNA extraction and quantitative PCR (qPCR) were published in our previous study (Sailaja et al., 2014). Total RNA from flag leaf of N22 and Vandana was isolated by using RNeasy Plant Mini Kit (Qiagen). cDNA synthesis of mRNAs was done using Improm-II reverse transcription system (Promega), and qRT-PCR was performed using SYBR Premix Ex-Taq (Takara). Actin was chosen as an internal control, and all the reactions were run in triplicate.

qPCR conditions for genes were 50°C for 10 min for preholding stage, 95°C for 10 min for holding stage, 40 cycles of denaturation at 95°C for 15 s, and annealing plus extension at 60°C for 30 s, followed by a disassociation stage (melt curve analysis). In order to analyze the real-time PCR data, the comparative threshold cycle (CT) method was used. The CT-values are provided in Supplementary File 2. ΔCT was calculated by CT target minus CT reference. ΔΔCT-values were calculated by ΔCT of treated sample minus ΔCT control sample. Fold difference of genes expression was calculated from 2-^Δ*ΔCt*^. To calculate, ΔCT standard deviation, we followed http://www3.appliedbiosystems.com/cms/groups/mcb_support/documents/generaldocuments/cms_042380.pdf. The positive value of ΔΔCT suggested down-regulation of transcript. Here, if the test sample had a value of 0.25, then it suggested 1/4 the amount of target RNA as the calibrator and was represented as 4.0-fold down-regulation.

## Results

### Cell membrane thermostability (MTS)

**Figure 2:**
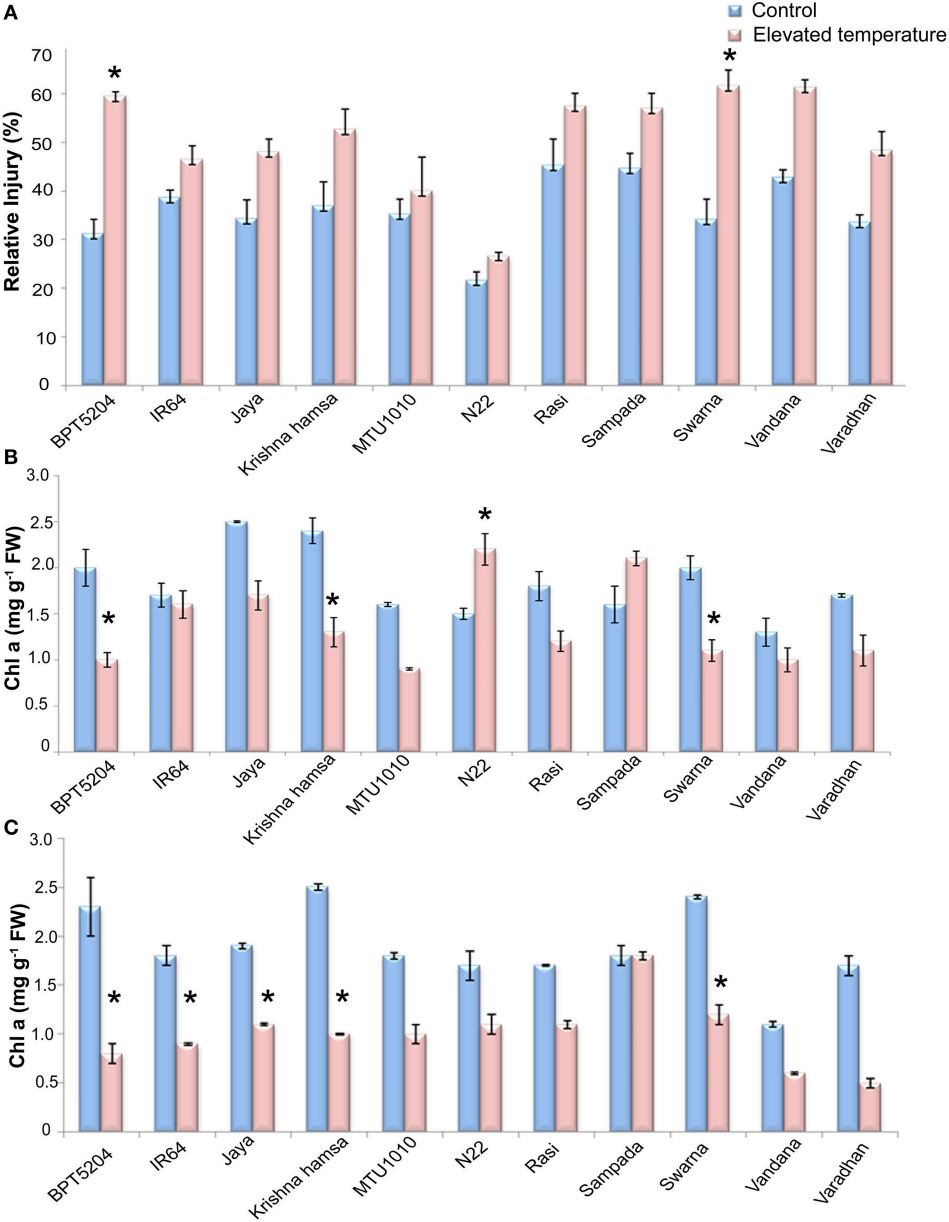
**Effect of elevated temperature on (A) Relative injury, (B) Chl a (vegetative phase), and (C) Chl a (reproductive phase) under control and elevated temperatures**. Each value represents mean of three replications ± SEm. Statistically significant values are shown using a star (^*^) in bar diagrams.

MTS at vegetative phase was analyzed in terms of relative injury (RI). Highly significant differences were observed between treatments and also between varieties (*P* < 0.01). Elevated temperature stress (ETS) increased the mean RI by 40% with respect to control. Highest increase in RI during ETS was observed in BPT5204 (90%) followed by Swarna (80%), Krishna Hamsa (42.4%), and Vandana (43%). On the other hand, minimum increase in RI (< 30%) during ETS was observed in MTU1010 (13%), IR64 (20%), N22 (23%), and Rasi (27%) (Figure 2A).

### Photosynthetic pigments

Photosynthetic pigments (*Chl a* and *Chl b*) and carotenoids were measured. Significant differences were observed in C*hl a* and *Chl* a/b, and total chlorophyll at ETS when compared with control. However, differences were not significant in case of *Chlb* and carotenoid content. Mean reduction of *Chl a* by 24 and 51% in vegetative and reproductive phases, respectively, was observed at ETS. During vegetative phase, increase in *Chl a* content was observed in N22 and Sampada, whereas maximum reduction was observed in BPT5204. At reproductive phase, reduction in *Chl a* was observed in almost all cultivars (Figures 2B,C). Furthermore, reduction in total chlorophyll content was also observed in all cultivars during vegetative and reproductive phases by a mean of 14.9 and 42%, respectively (Supplementary Tables 1, 2).

### Antioxidants

**Figure 3:**
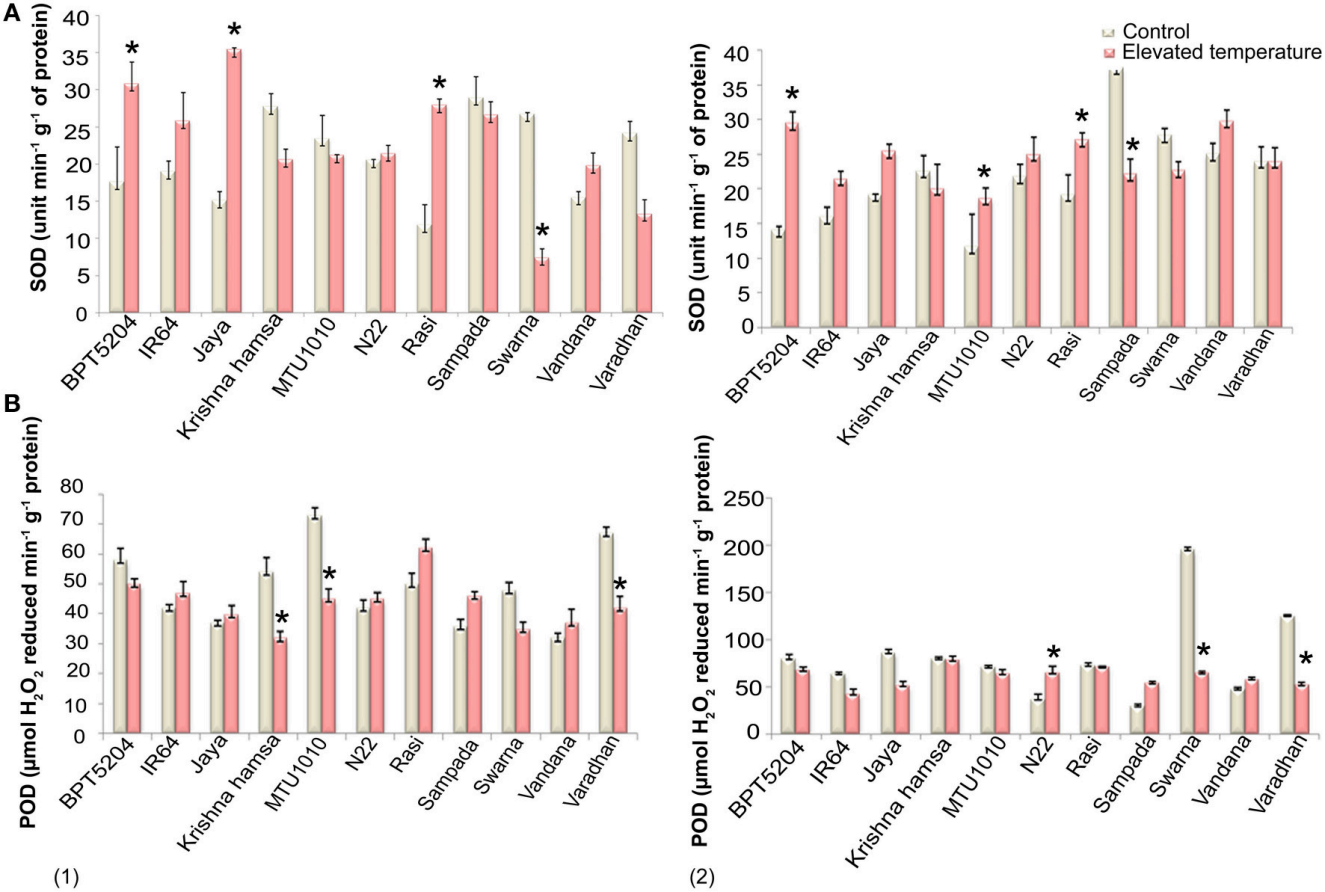
**Effect of elevated temperature on (A) SOD and (B) POD activity at (1) vegetative phase and (2) reproductive phase**. Each value represents mean of three replications ± SEm. Statistically significant values are shown using a star (^*^) in bar diagrams.

SOD, CAT, and POD activities were measured in control and ETS samples. Significant differences in SOD activity were observed during vegetative and reproductive phases under elevated temperature. Increased SOD activity was recorded in BPT5204, IR64, Jaya, N22, Rasi, and Vandana cultivars, whereas decreased SOD activity was noticed in Krishna Hamsa, Sampada, and Swarna at both the phases during elevated temperature (Figure 3A). Unlike SOD, significant differences in CAT activity were not observed at ETS. Although, N22 showed increased CAT activity at both vegetative and reproductive phases (Supplementary Table 3), POD activity was significantly affected under ETS during both the phases. Cultivars such as N22, Sampada, and Vandana showed increased POD activity in vegetative and reproductive phases (Figure 3B). Among 11 cultivars chosen for antioxidant enzymes activity assay, only N22 showed increased activity of each of the three enzymes (SOD, CAT, and POD) at both vegetative and reproductive stages under ETS.

### Photosynthesis

**Figure 4:**
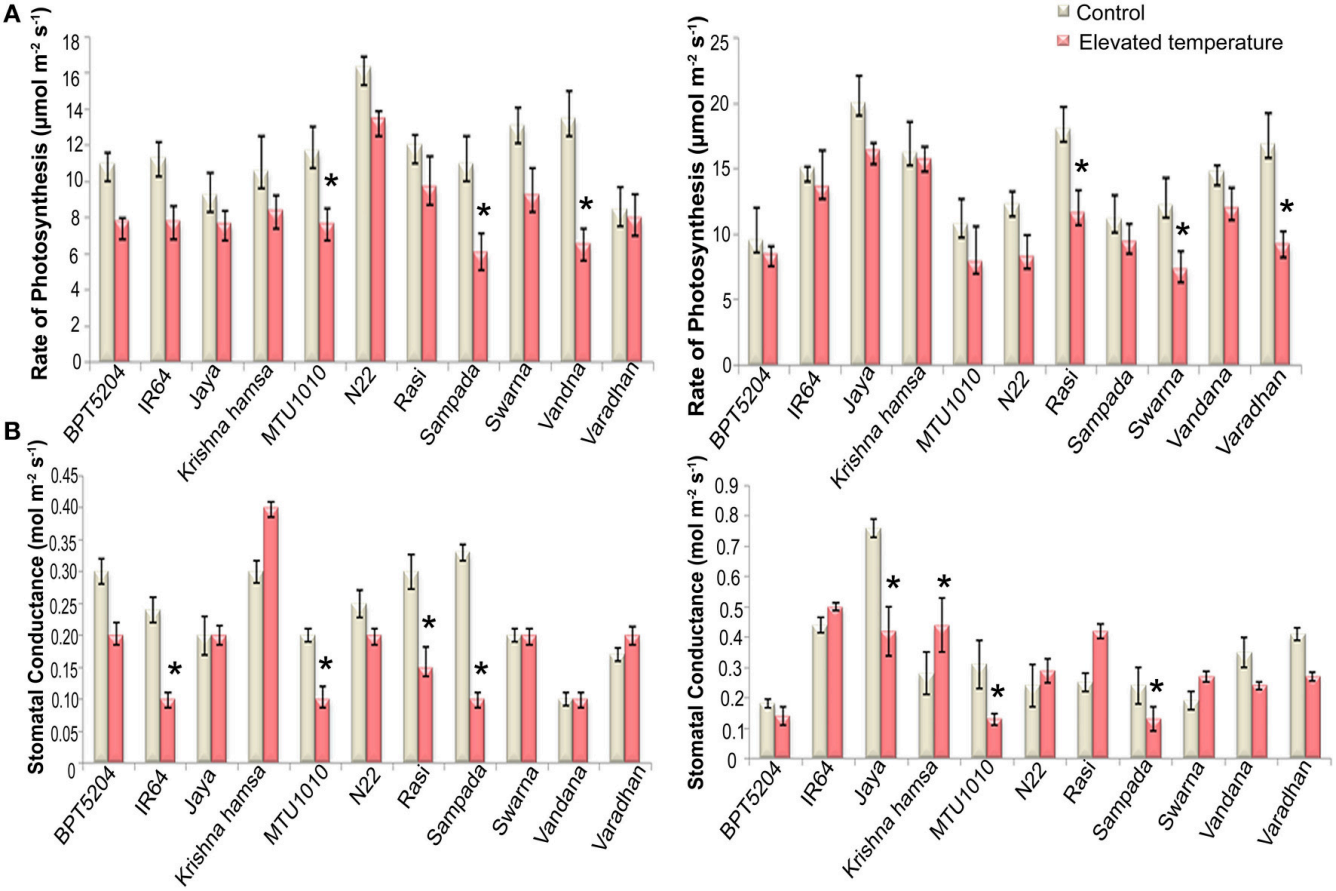
**Effect of elevated temperature on (A) Rate of Photosynthesis (*P*_*N*_) and (B) Stomatal Conductance(*g*_*s*_) at (1) vegetative phase (2) reproductive phase**. Each value represents mean of three replications ± SEm. Statistically significant values are shown using a star (^*^) in bar diagrams.

**Figure 5:**
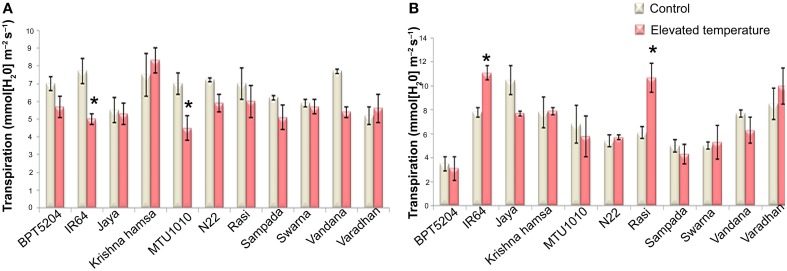
**Effect of elevated temperature on Transpiration Rate (*E*) at (A) vegetative phase and (B) reproductive phase**. Each value represents mean of three replications ± SEm. Statistically significant values are shown using a star (^*^) in bar diagrams.

To examine the effects of elevated temperature on photosynthesis, photosynthetic and fluorescence characters were measured in all cultivars at vegetative and reproductive phases. Different parameters such as net photosynthetic rate (*P*_*N*_), stomatal conductance (*g*_*s*_), transpiration rate (*E*), internal CO_2_ concentration (*C*_*i*_), ratio of intercellular and control CO_2_ (*C*_*i*_*/C*_*a*_), and water use efficiency (iWUE) were analyzed. Reduction in *P*_*N*_ was observed in all cultivars at both the phases under ETS. The mean *P*_*N*_ was significantly reduced by 27.8 and 23% at vegetative and reproductive phases, respectively. Significant differences were noticed among the varieties. The interaction between treatment and variety (T × V) was also found statistically significant (*P* < 0.01). Maximum reduction of *P*_*N*_ was observed in BPT5204, Vandana, and Varadhan, whereas minimum reduction of *P*_*N*_ was observed in Jaya, N22, Rasi, Krishna Hamsa, and IR64 at vegetative and reproductive phases (Figure 4A). The mean *g*_*s*_ and *E* were more significantly affected during vegetative phase than reproductive phase under ETS. The reduction in mean *g*_*s*_ was observed to the tune of 21.7% (vegetative) and 11% (reproductive) (Figure 4B). Jaya and Rasi showed significant increase in *E* at reproductive phase under elevated temperature (Figure 5). *Ci* and *C*_*i*_*/C*_*a*_ were not affected significantly at reproductive phase when compared with vegetative phase (Supplementary Table 4). Increased *Ci* was observed in IR64, Krishna Hamsa, N22, Rasi, Swarna, Vandana, and Varadhan, whereas increased *C*_*i*_*/C*_*a*_ ratio was observed in BPT5204, IR64, Jaya, Krishna Hamsa, N22, Rasi, Swarna, Vandana, and Varadhan under ETS at both the phases, but it was statistically non-significant. Unlike other parameters, iWUE was significantly affected in reproductive phase. Increased iWUE was observed in BPT5204, IR64, Jaya, MTU1010, N22, Rasi, Sampada, and Vandana at both the phases during ETS, but it was statistically non-significant (Supplementary Table 5).

### Fluorescence parameters

**Figure 6:**
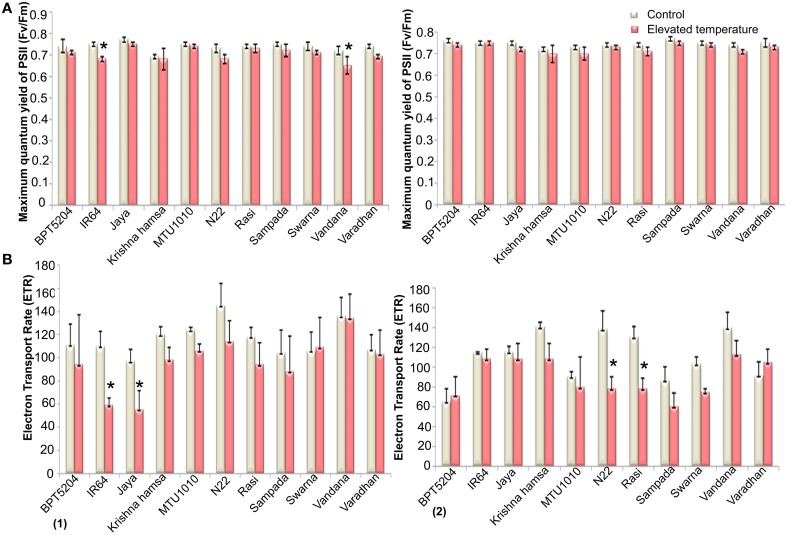
**Effect of elevated temperature on (A) Maximum quantum yield of PSII (Fv/Fm) and (B) Electron Transport Rate (ETR) at (1) vegetative phase (2) reproductive phase**. Each value represents mean of three replications ± SEm. Statistically significant values are shown using a star (^*^) in bar diagrams.

Along with the photosynthetic characteristics, different fluorescence parameters such as maximum quantum yield of PSII (Fv/Fm), efficiency of excitation capture by open PSII centers (Φe = Fv′/Fm′), electron transport rate (ETR), *in vivo* quantum yield of PSII photochemistry (Φ_PSII_), quantum yield of CO_2_ assimilation (Φ_CO2_), coefficient of photochemical quenching (*qP*), and co-efficient of non-photochemical quenching (*qN*) were measured. Marginal reduction of Fv/Fm ratio was observed in all cultivars at both the phases under ETS (Figure 6A). Maximum reduction of Fv/Fm was observed in Vandana and Rasi. Reduction of Fv′/Fm′ was also observed under ETS in all cultivars. Here, the reduction was more significant in reproductive phase when compared with vegetative stage. Four cultivars—IR64, Jaya, MTU1010, and N22—showed minimum reduction of Fv′/Fm′ (Supplementary Table 6). The mean ETR of all the cultivars was decreased by 16.7 and 19% at vegetative and reproductive phases, respectively, under ETS (Figure 6B).

Reduction in Φ_PSII_ and Φ_CO2_ was observed in all the cultivars during ETS at vegetative and reproductive phases. Furthermore, more significant reduction (21%) of Φ_PSII_ was observed at reproductive phase. Mean reduction in *qP* by 12 and 15% was observed at vegetative and reproductive phases, respectively. Marginal increase in *qP* was observed in Varadhan, whereas in other cultivars, *qP* was reduced under ETS. Increase in *qN* was observed in maximum cultivars during vegetative and reproductive phases under ETS. At reproductive stage, decrease in *qN* was observed in BPT5204 and Varadhan, whereas other cultivars showed increased *qN* under ETS (Supplementary Table 7).

### Plant height, tiller number, and number of panicles per hill

Plant height at maturity was measured at ETS and control. Maximum increase in plant height at elevated temperature was observed in N22, whereas maximum decrease was observed in Varadhan. Significant differences in tiller number/panicle number (*P* < 0.05) were observed under ETS. Here, maximum increase was observed in Krishna Hamsa, whereas maximum decrease was observed in Jaya and BPT5204 (Supplementary Table 8).

### Yield attributes

The following yield-associated linked traits were recorded in rice cultivars grown at control and elevated temperatures.

### Days to 50% flowering and days to maturity

Observation on days to 50% flowering and days to maturity is presented in Table 2. There was significant reduction in number of days to 50% flowering in all cultivars under ETS. In addition, significant decrease in number of days to physiological maturity was observed in all cultivars under ETS. The grain-filling period (the difference between flowering and maturity) was also decreased under elevated temperature in almost all cultivars. The mean of grain-filling period for all varieties was reduced by 2 days. Maximum reduction of 6 days was observed in Krishna Hamsa and Rasi followed by IR64 and N22 (Table 2).

**Table 2:** **Effect of elevated temperature on days to 50% flowering, maturity, and grain-filling period**.

**Cultivar**	**Days to 50% flowering**		**Days to maturity**		**Grain-filling period (days)**	
	**Control**	**ETS**	**Control**	**ETS**	**Control**	**ETS**
BPT5204	113	110	136	132	23	22
IR64	84	80	112	105	28	25
Jaya	94	90	122	118	28	28
Krishna Hamsa	82	78	124	114	42	36
MTU1010	84	81	114	109	30	28
N22	67	63	97	90	30	27
Rasi	80	78	120	112	40	34
Sampada	109	107	132	128	23	21
Swarna	120	116	145	139	25	23
Vandana	63	57	89	82	26	25
Varadhan	86	83	112	108	26	25
Mean	89	86	118	112	29	26

### Number of filled grains per hill, spikelet sterility, 1000 grain weight, and grain yield/hill

**Figure 7:**
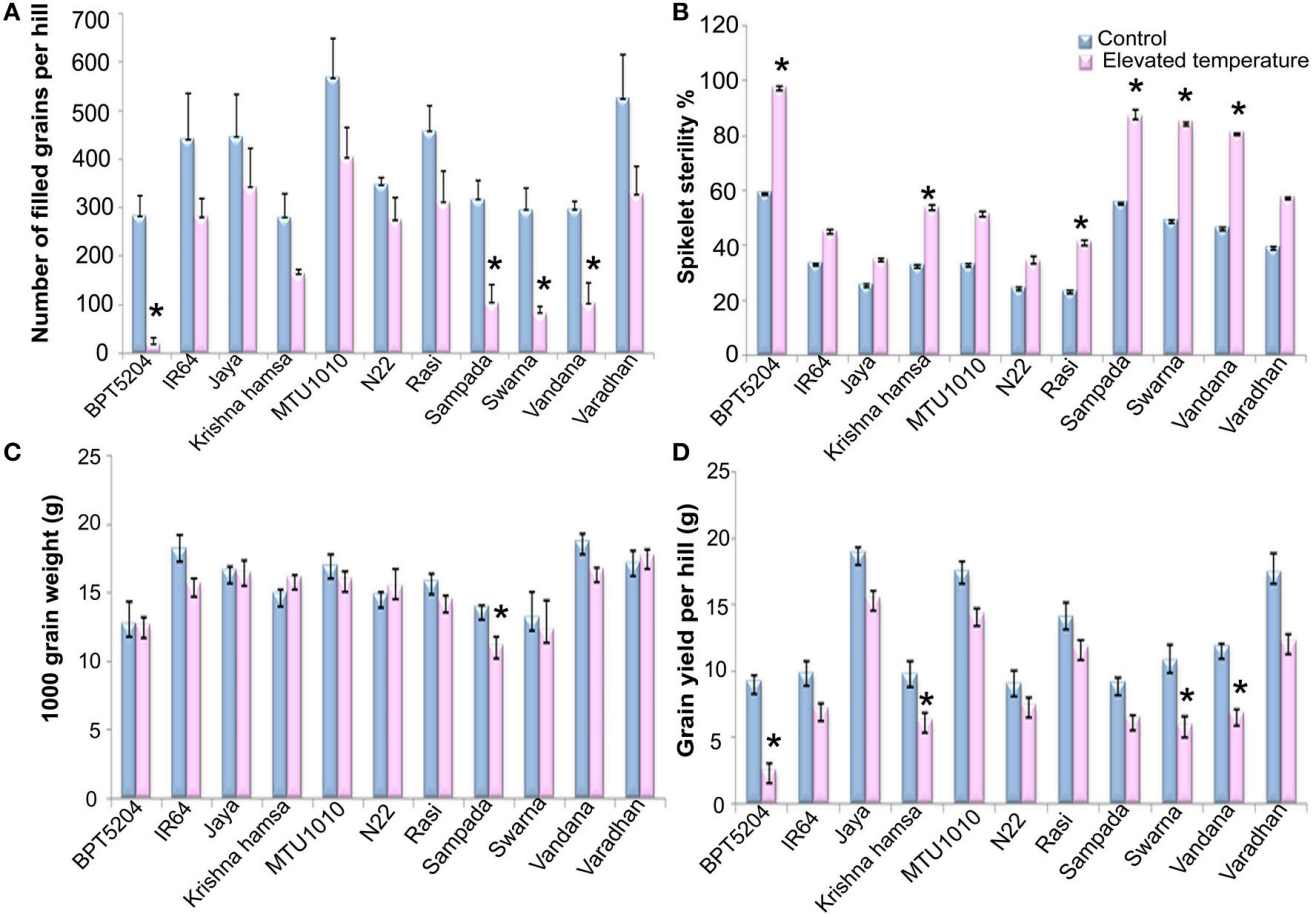
**Effect of elevated temperature on (A) Filled grain number per hill, (B) Spikelet sterility %, (C) 1000 grain weight, and (D) Total grain yield per hill in different rice cultivars**. Each value represents mean of three replications ± SEm. Statistically significant values are shown using a star (^*^) in bar diagrams.

Exposure to elevated temperature significantly affected (*P* < 0.01) filled grain number per hill. It was reduced by 41% under ETS in comparison with control. Number of filled grains varied significantly among the cultivars (*P* < 0.01). Maximum reduction of filled grain number was observed in BPT5204 (93% over control) followed by Vandana (72%), Sampada (67%), and Swarna (65%). On the contrary, minimum reduction was observed in N22 (20%), Jaya (23%), and MTU1010 (28%) (Figure 7A). Elevated temperature showed significant negative impact on spikelet fertility. BPT5204 (97.3%), Sampada (86.7%), Swarna (84.7%), and Vandana (80.9%) showed maximum spikelet sterility, whereas it was minimum in N22 (34.3%), Jaya (35.1%), MTU1010 (34.3%), and Rasi (41%) (Figure 7B).

Significant reduction of 5% in 1000 grain weight was observed at ETS (*P* < 0.01). Differences among the varieties and T × V interaction were also found to be significant (*P* < 0.01). BPT5204, Jaya, N22, Swarna, and Varadhan showed no significant difference, whereas reduction was observed in IR64, Sampada, and Vandana under ETS. Increase in 1000 grain weight was observed in Krishna Hamsa during elevated temperature (Figure 7C). Mean reduction of 29.4% in grain yield/hill (*P* < 0.01) was observed during ETS. Grain yield ranged between 5.9 g (BPT5204) and −17.2 g (Jaya) per hill. Maximum reduction in grain yield under ETS was observed in BPT5204 (72%) followed by Swarna, Sampada, and Vandana, whereas minimum reduction was observed in N22, Jaya MTU1010, and Rasi when compared with control (Figure 7D).

### Correlation analysis

To observe the correlation between yield attributes and different biochemical/physiological traits studied under ETS, a multiple correlation analysis was performed (Supplementary Table 9). The correlation coefficient values indicated that spikelet fertility was positively and significantly associated with grain yield recorded under ETS. Furthermore, a strong negative association of relative injury % and maximum quantum yield of PSII (Fv/Fm) was observed with grain yield under ETS. *E* (transpiration rate) at reproductive stage also showed a positive association with grain yield under heat stress.

### Genes expression

To study the molecular response, expression of 14 genes was analyzed in representative susceptible and tolerant rice genotypes. N22 was selected as tolerant, whereas Vandana was selected as susceptible genotype based on the physiological, biochemical, and yield studies. These two genotypes were selected for gene expression study, as they have similar flowering time and maturity duration. The expression analysis was done at reproductive phase considering it as more sensitive to stress. In order to analyze gene expression, heat stress treatment (42°C for 24 h) was given at flowering stage in a controlled environment (plant growth chamber). In our previous study (Sailaja et al., 2014), 13 genes were used to study the heat stress response in young seedlings of rice. Here, in addition to those 13 genes, *FRH* was also included for gene expression analysis at reproductive phase.

**Figure 8:**
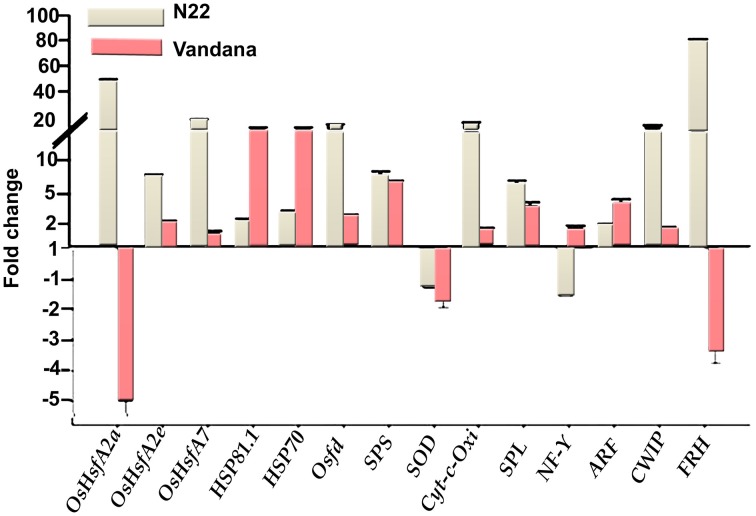
**Expression analysis of genes in N22 and Vandana cultivars at reproductive phase**. The fold change expression was calculated by comparing the expression level at control and high temperature. Actin was chosen as internal control.

N22 showed very high expression of heat shock transcription factors—*OsHsfA2a, OsHsfA2e*, and *OsHsfA7*—to the tune of 49.0-, 6.1-, and 17.3-fold under heat stress with respect to control. Vandana also showed upregulation of *OsHsfA2e* and *OsHsfA7*, although the degree of expression was very less compared to N22. *OsHsfA2a* was down-regulated in Vandana. The other highly upregulated genes in N22 during heat stress were *Osfd* (13.7-fold), *Cyt-C-Oxi* (14.2-fold), *CWIP* (12.5-fold), and *FRH* (80.0-fold). In Vandana also, *Osfd, Cyt-C-Oxi*, and *CWIP* showed upregulation under heat stress, but the expression was very less compared to N22. However, *FRH* was down-regulated in Vandana. Heat shock protein genes *HSP81.1* and *HSP70* showed increased expression in both the cultivars under heat stress, although expression was more in Vandana. *SPS, SPL*, and *ARF* were upregulated, whereas *SOD* was down-regulated in both the genotypes during stress (Figure 8).

## Discussion

Rice, being the most important crop to ensure food security, faces challenges of climate change. Global warming is one of the most serious concerns in changing climatic conditions, which has direct impact on agriculture. Increase in temperature will lead to heat stress, which affects yield and quality of agricultural crops. Although, several studies have been performed to understand the effect of heat stress in crops and particularly in rice (Oh-e et al., 2007; Jagadish et al., 2008; Shi et al., 2014), most of these studies were based on exposing the plants to high temperature for limited duration at either vegetative or reproductive phases. Keeping in view that global mean temperature is rising, it is important to understand the response of plants exposed to high temperature throughout their growth phase, particularly from flowering stage till seed maturity. It will not only help in understanding the adaptation plasticity of different genotypes to high temperature but also provide information on identifying the important traits for breeding of genotypes suitable to these environmental conditions. In this study, 11 rice varieties grown widely in India were selected. These genotypes were grown in control and elevated temperatures from seedling to maturity phase. Important physiological and biochemical processes were studied to understand the differential response of these rice varieties to elevated temperature at vegetative and reproductive phases.

In rice, reproductive stage is the most sensitive stage to heat (Yoshida et al., 1981) and anthesis/flowering is the most severely affected process (Satake and Yoshida, 1978; Nakagawa et al., 2002; Jagadish et al., 2008; Shi et al., 2014). In general, high temperature is unfavorable for flowering and grain filling by causing spikelet sterility and shortening the duration of grain-filling phase (Tian et al., 2007; Xie et al., 2009). High temperature during ripening stage generally reduces grain weight and grain-filling phase, and increases the percentage of white chalky rice (Osada et al., 1973; Yoshida et al., 1981). At elevated temperature, decrease in number of days to 50% flowering and days to grain maturity was observed in all cultivars. Similarly, grain-filling phase was also reduced in almost all cultivars. High temperature showed significant impact on yield through sharp reduction of filled grain number/hill and increased sterile spikelets. Maximum filled grain reduction was observed in BPT5204, Sampada, Swarna, and Vandana. On the other hand, minimum reduction in filled grain number was observed in N22, Jaya, MTU1010, and Rasi at elevated temperature. Similar to reduction in filled grain number/hill, spikelet sterility was observed more in BPT5204, followed by Swarna, Vandana, and Sampada at ETS. High temperature leads to spikelet sterility due to poor anther dehiscence and low pollen production (Matsui et al., 1997; Prasad et al., 2006). In addition, significant reduction of 1000 grain weight and grain yield per hill was also observed at ETS. Based on yield attributes at ETS, these rice cultivars were categorized as tolerant, moderately tolerant, and susceptible.

In order to understand the important physiological and biochemical phenomena contributing to differential elevated temperature response, various parameters such as MTS, antioxidant enzymes, chlorophyll and carotenoid contents, and photosynthesis and fluorescence characteristics were measured in all the genotypes at vegetative and reproductive phases. Among the physiological and biochemical traits studied here, MTS was the most reliable trait that showed maximum correlation with yield attributes under ETS. When the difference of RI in ETS and control samples was observed, four cultivars showed < 30% increase in RI, i.e., 13% increase in MTU1010, 20% in IR64, 23% in N22, and 17% in Rasi. These cultivars showed good performance in yield attributes also. Earlier studies showed that plants with higher electrolyte leakage/relative injury found to be more susceptible toward high temperature stress (Reynolds et al., 1994; Haque et al., 2009). Studies in rice also showed that heat-tolerant genotypes possess better membrane integrity than heat-sensitive ones (Mohammed and Tarpley, 2009; Kumar et al., 2012). Interestingly, Jaya showed high RI even though it showed less reduction in filled grain percentage, suggesting that it may have different mechanism to cope with high temperature stress. Jaya showed increased SOD activity at both the stages (vegetative and reproductive) and minimum reduction of *P*_*N*_ and Fv′/Fm′ under ETS. SOD and Fv′/Fm′ play crucial role in stress response in plants (Kumar et al., 2012; Sharma et al., 2012).

Increased antioxidant activity under heat is a general response shown by plants (Wahid et al., 2007). In this study also, increased SOD and POD activities were observed in susceptible as well as in tolerant genotypes. Mohammed and Tarpley (2009) showed negative association of *Chla*, total chlorophyll content, and *Chl a/b* with high temperature in rice, but *Chl b* was not affected, which was observed in this study too. Here, significant difference in *Chla, Chl a/b*, and total chlorophyll was observed between control and ETS at reproductive phase; however, *Chlb* and carotenoid content were not affected significantly. Retention of chlorophyll for greater duration under high temperature was reported in tolerant genotypes of rice and creeping bentgrass (*Agrostis palustris* Huds; Sohn and Back, 2007). Xie et al. (2012) reported that high air temperature during heading stage negatively influenced SPAD-value (relative content of chlorophyll) in rice flag leaves.

In plants, photosynthesis is one of the most susceptible processes to high temperature stress (Yin et al., 2010). Considerable reduction of photosynthesis during high temperature stress in rice leaves was reported (Taniyama et al., 1988). Cao et al. (2009) reported that high temperature during maximum vegetative stage and early grain-filling phases caused a reduction in photosynthetic rate of flag leaf in different rice cultivars. In this study, photosynthetic parameters such as *P*_*N*_, *g*_*s*_, *E, Ci*, and iWUE were analyzed in vegetative and reproductive phases of 11 cultivars grown at control and ETS. These parameters were significantly affected, suggesting that photosynthesis is highly sensitive to high temperature in rice genotypes. While analyzing the gas exchange parameters such as *g*_*s*_, *E*, and *Ci*, it was observed that the effect of ETS was more pronounced during vegetative phase for these traits in particular. Egeh et al. (1992) reported that higher *E, g*_*s*_, and *Ci* contribute to high temperature tolerance. Increase in *E* and higher *g*_*s*_ result in considerable lowering of leaf and canopy temperature, which reduces the harmful effect of high temperature. Although photosynthetic parameters were significantly influenced by high temperature in this study, they did not show distinct correlation with yield attributes of susceptible and tolerant rice cultivars, thereby suggesting that it may be a general physiological response of plants to high temperature.

In stress physiology, chlorophyll fluorescence is another important technique to evaluate the damage of leaf photosynthetic apparatus, in particular PSII activity (Maxwell and Jhonson, 2000; Baker and Rosenqvist, 2004). It was used to assess the genetic variability for heat stress tolerance in wheat (Sharma et al., 2012, 2014). In this study, elevated temperature showed marginal reduction in maximum quantum yield of PSII (Fv/Fm) during vegetative as well as reproductive phases. Fv/Fm-values did not show much reduction at elevated temperature in comparison to control, which may be due to the gradual adaptation of plants to elevated temperature conditions. Significant reduction of Fv′/Fm′ (Φe efficiency of excitation capture by open PSII centers) was noticed in all genotypes under ETS at both the phases. Furthermore, Φ_PSII_, *qP*, and ETR were also significantly reduced, whereas *qN* was increased. Song et al. (2014) reported reduction in *qP* and ETR at high temperature (42°C) in populus. Reduction in ETR under high temperature stress is due to inactivation of oxygen-evolving complex (OEC) (Luo et al., 2011) and less utilization of NADPH and ATP under reduced photosynthesis (Lu and Zhang, 1999; Subrahmanyam and Rathore, 2000).

Correlation analysis suggests that MTS and *E* are the most useful parameters to phenotype for ETS tolerance. RI showed a strong negative association, whereas *E* at reproductive phase showed positive association with grain yield. IR64 (11.1 ± 0.6) and Rasi (10.7 ± 1.2) showed high *E* under elevated temperature. Furthermore, *g*_*s*_ and *E* showed significant and positive association with filled grain number. High *g*_*s*_ and *E* might be reducing canopy temperature that causes lower panicle temperature and facilitate increase in spikelet fertility under ETS. Reduction in panicle micro-climate temperature by surrounding leaves reduces the effect of high temperature (Shi et al., 2014). This study suggests that different genotypes may have evolved different mechanisms to develop ETS tolerance, which signifies the complexity of pathways associated with high temperature response. Important biochemical and physiological traits contributing to ETS tolerance need to be characterized in individual genotypes to facilitate the breeding of climate-resilient rice genotypes. From this study, we have listed the useful traits identified in 11 genotypes, which can be used to develop elevated temperature-tolerant rice cultivars (Table 3).

**Table 3:** **Physiological and biochemical traits identified in 11 popular rice genotypes under elevated temperature stress**.

**Traits**	**Vegetative phase**	**Reproductive phase**
RI	Less in MTU1010, IR64, N22, and Rasi (≤30%)	
*Chl a*	Increased in N22 (44%) and Sampada (34%)	Increased in Sampada (3%)
*Chl b*	Increased in Vandana (195%), Swarna (167%), N22 (43%), Varadhan (27%), and IR64 (9%)	Increased in MTU1010 (62%), Varadhan (54%), Sampada (38%), and IR64 (8%)
Total *Chl*	Increased in N22 (44%), Vandana (29%), and Sampada (23%)	Reduction was observed in all varieties but minimum in Krishna Hamsa followed by N22
SOD	Maximum increase in Rasi (135%) and Jaya (134%)	Maximum increase in BPT5204 (109%), MTU1010 (60%), Rasi (41%), IR64 (34%), and Jaya (32%)
CAT	Increased in Swarna (78.8%), BPT5204 (57%), and Sampada (37.31%)	Increased in Varadhan (175%), N22 (117%), and IR64 (35%)
POD	Increased in Sampada (27%), Rasi (23%), Vandana (14%), IR64 (11%), N22 (8%), and Jaya (7%)	Increased in Sampada (83%), N22 (75%), and Vandana (21%)
Net photosynthetic rate (*P_*N*_*)	Minimum reduction in Jaya (−16%), N22 (−17%), and Rasi (−18%)	Minimum reduction in Krishna Hamsa (−2%), and IR64 (−4%)
Stomatal conductance (*g_*s*_)*	Increased in Krishna Hamsa (42%), Varadhan (24%), and Jaya (5%)	Increased in Rasi (70%), Swarna (43%), Krishna Hamsa (55%), IR64 (24%), and N22 (22%)
Transpiration (*E*)	Increased in Krishna Hamsa (10%) and Varadhan (7%)	Increased in Rasi (4.5%) and IR64 (3%)
*C_*i*_*	Increased in Vandana (28%), Krishna Hamsa (9%), and Varadhan (3%)	Increased in Varadhan (23%), IR64 (19%), Swarna (18%), Krishna Hamsa (16%), Rasi (11%), and N22 (6%)
*C_*i*_/Ca*	Increased in Vandana (28%), Krishna Hamsa (9%), Jaya (4%), and Varadhan (4%)	Increased in Varadhan (21%), IR64 (19.25%), Krishna Hamsa (15%), Swarna (17%), Rasi (10%), and N22 (5%)
*P_*N*_/g_*s*_*	Increased in IR64, MTU1010, N22, Rasi, and Sampada	Increased in BPT5204, Jaya, MTU1010, Sampada, and Vandana
Fv/Fm	Minimum reduction in MTU1010 and Krishna Hamsa	Significant difference was not observed
Fv′/Fm′	Minimum reduction in N22 (−1%), IR64 (−4.2%), and MTU1010 (−4.4%)	Minimum reduction in Sampada (−2%), IR64 (−3%), and Varadhan (−3%)
Φ_PS2_	Minimum reduction in Jaya, Krishna Hamsa, and Vandana	Marginal increase in Varadhan
Φ_Co2_	Minimum reduction Krishna Hamsa, Swarna, and Varadhan	Marginal increase in Sampada and Varadhan
*qP*	Significant differences were not observed among cultivars	Marginal increase in Varadhan
*qN*	All showed increased *qN* except IR64 and Varadhan	All showed increased *qN* except BPT5204 and Varadhan

Interestingly, N22 emerged as the most tolerant genotype to elevated temperature. It has been categorized as heat tolerant in earlier studies (Egeh et al., 1992; Ziska et al., 1996; Jagadish et al., 2008). Interestingly, this aus genotype shows tolerance to high temperature irrespective of the treatments, i.e., short duration exposure, long duration exposure, or continuous high temperature treatment (in this study), which is not shown by other genotypes studied so far. Furthermore, it shows tolerance character at all stages of crop growth, i.e., seedling, vegetative, and reproductive phases (Jagadish et al., 2008; Krishnan et al., 2011; Sailaja et al., 2014). N22 showed best performance in almost all the parameters studied here at vegetative and reproductive phases, e.g., lesser RI (23%), lesser reduction in chlorophyll content, increased SOD, CAT, and POD activities, lesser reduction in *P*_*N*_, and high transpiration rate causing minimum reduction in spikelet fertility and grain yield under ETS. This was further supported with the gene expression analysis of N22, showing very high expression of heat shock transcription factors (*OsHsfA2a, OsHsfA2e*, and *OsHsfA7*) during high temperature stress at flowering stage. Hsfs are important transcriptional regulatory proteins of plants playing key role in controlling the expression of several heat-responsive genes (Qiao et al., 2015). Overexpression of *Hsf* genes in transgenic plants resulted in upregulation of heat stress-associated genes and an enhancement of thermotolerance (Mishra et al., 2002; Charng et al., 2007; Yokotani et al., 2008). Induced expression of *OsHsfA2a, OsHsfA2e*, and *OsHsfA7* during heat stress was reported in rice (Wang et al., 2009). *Osfd, Cyt-C-Oxi*, and *CWIP* also showed very high expression in heat stressed tissues of N22. Significant increase in expression of *Osfd* and *CWIP* in N22 was reported in our previous study where heat stress was applied in seedling stage (Sailaja et al., 2014). The annotation of *Osfd* suggests it as iron–sulfur cluster-binding protein involved in electron transport activity and is located in chromosome1 (locus Os01g0730500). Another highly upregulated gene during heat stress in N22 was *FRH*, which is annotated as fertility restorer homolog A, a prenyltransferase domain containing protein. In comparison to N22, the susceptible cultivar Vandana showed very less expression of *Hsfs, Osfd, Cyt-C-Oxi*, and *CWIP*, whereas *FRH* was down-regulated. It would be interesting to further characterize their functional role in high temperature stress tolerance. A multiple organizational level analysis including physiological, biochemical, and transcriptional responses suggested N22 as the most efficient heat-tolerant genotype in this study.

In summary, this is an important study where popular and widely grown rice genotypes were characterized for heat stress response by growing plants at continuous high temperature proportional to ambient temperature during the whole period of study. Several important physiological and biochemical traits were identified in different genotypes, which would be useful in phenotyping and breeding for heat stress tolerance. This study emphasizes that individual genotypes need to be characterized for specific heat stress treatments as it is a complex phenomenon where different genotypes have evolved different ways to respond to elevated temperature. Our study demonstrates that N22 is highly suitable to high temperature, showing best expression of useful genes and physiological and biochemical traits that can be utilized in breeding programs for high temperature tolerance.

The identified physiological and biochemical traits imparting heat stress tolerance in different rice genotypes can be genetically mapped and introgressed into heat-susceptible, high-yielding rice genotypes through breeding. Alleles of differentially expressed genes and their promoters/regulators can be sequenced from susceptible and tolerant rice genotypes to identify polymorphic genetic loci linked with stress tolerance. Such genetic loci can be validated in heat stress tolerance in a set of susceptible and tolerant rice genotypes and in mapping populations. The molecular markers based on these genetic loci could be directly useful in selecting heat tolerant genotypes through marker-assisted breeding. Furthermore, resistant or tolerant alleles of such genes can be used for genetic transformation of high-yielding but heat-susceptible genotypes.

## Author contributions

SM, DS, SN, SV, and VB designed the research, BS, TV, YR, and PV performed the research, BS, DS, and SM analyzed the data, SM and BS wrote the manuscript. All authors read and approved the manuscript.

### Conflict of interest statement

The authors declare that the research was conducted in the absence of any commercial or financial relationships that could be construed as a potential conflict of interest.

## Supplementary material

Supplementary Figure 1 **Maximum and minimum temperature recorded inside and outside the polyhouse from transplantation to maturity of rice genotypes**.

## Abbreviations

N22: Nagina22
ETS: elevated temperature stress
RI: relative injury
SOD: superoxide dismutase
CAT: catalase
POD: peroxidase
*P*_*N*_: net photosynthetic rate
*g*_*s*_: stomatal conductance
*E*: transpiration rate
*C*_*i*_: internal CO_2_concentration
iWUE: water use efficiency
Fv/Fm: quantum yield of PSII
Fv′/Fm′: efficiency of excitation capture by open PSII centers
ETR: electron transport rate
Φ_PSII_: *in vivo* quantum yield of PSII photochemistry
Φ_CO2_: quantum yield of CO_2_ assimilation
qP: coefficient of photochemical quenching
qN: co-efficient of non-photochemical quenching.
