# Supplementary figures and images for: Integrated Physiological, Biochemical, and Molecular Analysis Identifies Important Traits and Mechanisms Associated with Differential Response of Rice Genotypes to Elevated Temperature
Source: Front Plant Sci. 2015 Nov 27;6:1044. doi: 10.3389/fpls.2015.01044 (PMC4661239; doi:10.3389/fpls.2015.01044)

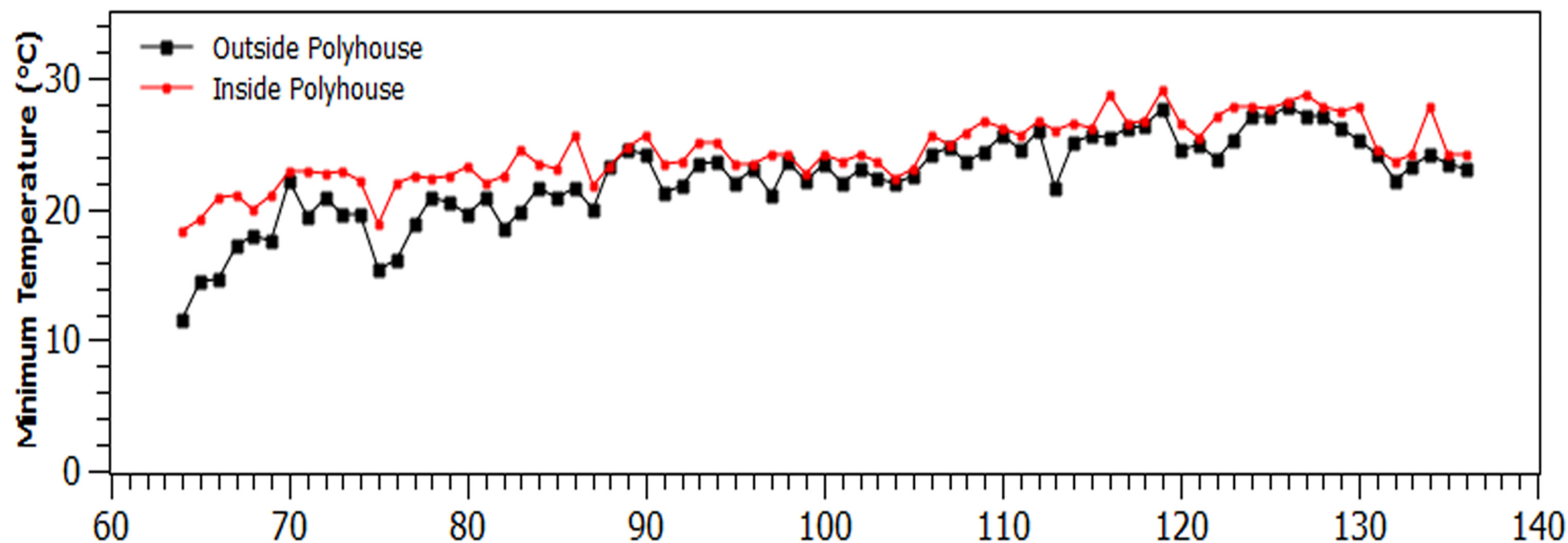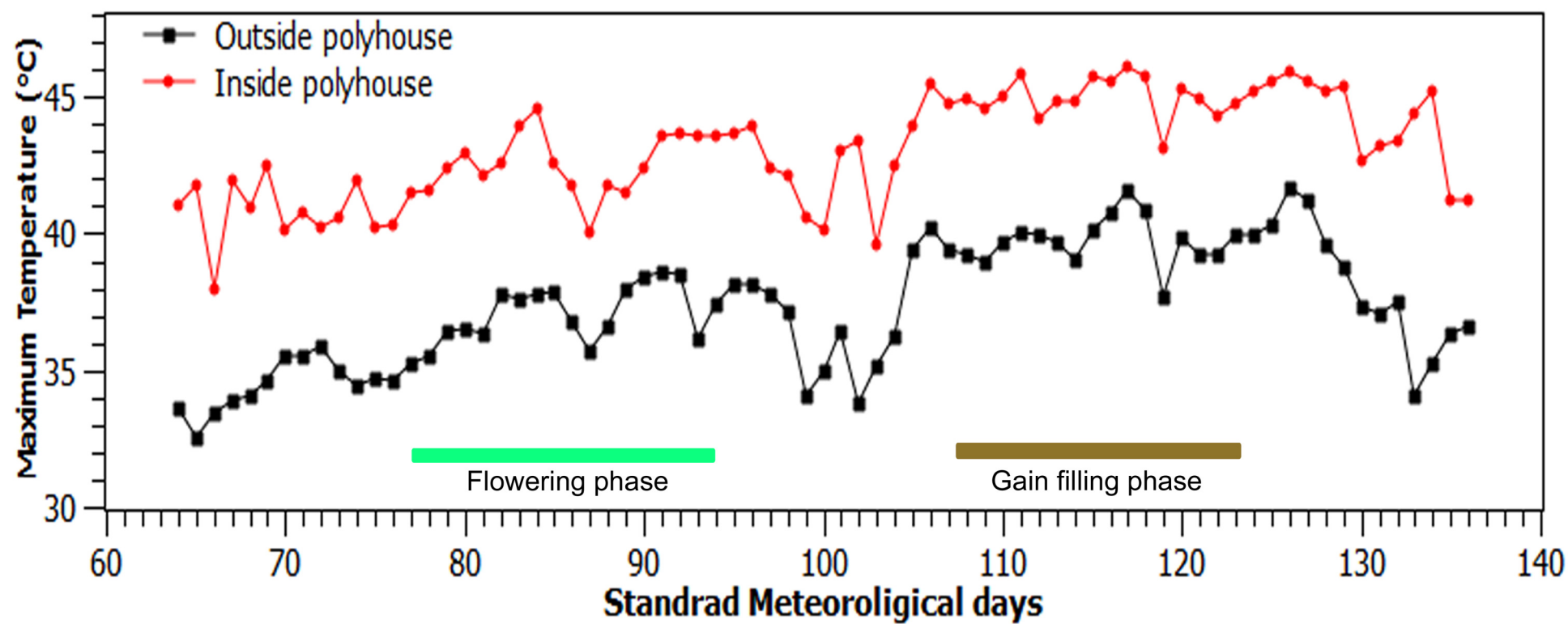

Supplement: Supplementary Figure 1 — Maximum and minimum temperature recorded inside and outside the polyhouse from transplantation to maturity of rice genotypes. [file Image1.PDF]
