# Supplementary material for: Integrated Physiological, Biochemical, and Molecular Analysis Identifies Important Traits and Mechanisms Associated with Differential Response of Rice Genotypes to Elevated Temperature
Source: Front Plant Sci. 2015 Nov 27;6:1044. doi: 10.3389/fpls.2015.01044 (PMC4661239; doi:10.3389/fpls.2015.01044)
Supplement: Supplementary file 2 [file Table1.DOC]

**Supplementary Table 1: Effect of elevated temperature on (1) Chl b and (2) T**otal chlorophyll at maximum vegetative stage and reproductive stage in selected rice cultivars.

| **(1)** | **Chl b( mg g^-1^ FW)** | | | | | |
| --- | --- | --- | --- | --- | --- | --- |
|  | Vegetative Stage | | | Reproductive Stage | | |
|  | Control | Elevated | Mean | Control | Elevated | Mean |
| BPT5204 | 0.5 ± 0.05 | 0.2 ± 0.06 | 0.4 | 0.7 ± 0.1 | 0.63± 0.03 | 0.5 |
| IR64 | 0.6 ± 0.02 | 0.7 ± 0.17 | 0.7 | 0.5 ± 0.2 | 0.5 ± 0.1 | 0.5 |
| Jaya | 0.7 ± 0.01 | 0.5 ± 0.04 | 0.6 | 0.4 ± 0.1 | 0.04 ± 0.01 | 0.2 |
| Krishnahamsa | 0.7 ± 0.18 | 0.4 ± 0.02 | 0.5 | 0.5 ± 0.05 | 0.3 ± 0.04 | 0.4 |
| MTU1010 | 0.4 ± 0.04 | 0.3 ± 0.02 | 0.4 | 0.3 ± 0.05 | 0.5 ± 0.03 | 0.4 |
| N22 | 0.4 ± 0.08 | 0.5 ± 0.27 | 0.5 | 1 ± 0.01 | 0.7 ± 0.1 | 0.8 |
| Rasi | 0.5 ± 0.07 | 0.3 ± 0.13 | 0.4 | 0.5 ± 0.01 | 0.3 ± 0.002 | 0.4 |
| Sampada | 0.6 ± 0.31 | 0.6 ± 0.1 | 0.6 | 0.5 ± 0.3 | 0.7 ± 0.001 | 0.6 |
| Swarna | 0.5 ± 0.05 | 1.4 ± 0.84 | 1 | 0.7 ± 0.02 | 0.6 ± 0.1 | 0.6 |
| Vandana | 0.4 ± 0.1 | 1.1 ± 0.18 | 0.7 | 0.7 ± 0.02 | 0.5 ± 0.004 | 0.6 |
| Varadhan | 0.9 ± 0.35 | 1.2 ± 0.06 | 1.1 | 0.3 ± 0.04 | 0.4 ± 0.1 | 0.4 |
| **Mean** | 0.6 | 0.7 | 0.6 | 0.5 | 0.46 |  |
| HSD(Treat) | 0.2(NS) | | | 0.05(P<0.01) | | |
| HSD(Variety) | 0.82(P<0.1) | | | 0.20(P<0.01) | | |
| HSD(T x V) | 1.31(NS) | | | 0.33(P<0.01) | | |

| **(2)** | **Total chlorophyll( mg g^-1^ FW)** | | | | | |
| --- | --- | --- | --- | --- | --- | --- |
|  | Vegetative Stage | | | Reproductive Stage | | |
|  | Control | Elevated | Mean | Control | Elevated | Mean |
| BPT5204 | 2.6 ± 0.28 | 1.2 ± 0.14 | 1.9 | 3 ± 0.4 | 1.4± 0.3 | 2.4 |
| IR64 | 2.3 ± 0.32 | 2.3 ± 0.28 | 2.3 | 2.3 ± 0.3 | 1.4 ± 0.1 | 1.9 |
| Jaya | 3.2 ± 0.02 | 2.2 ± 0.2 | 2.7 | 2.3 ± 0.5 | 0.8 ± 0.01 | 1.5 |
| Krishna hamsa | 3.1 ± 0.58 | 1.7 ± 0.39 | 2.4 | 2.9 ± 0.1 | 1.3 ± 0.1 | 2.1 |
| MTU1010 | 2.1 ± 0.07 | 1.1 ± 0.03 | 1.6 | 2.1 ± 0.2 | 1.5 ± 0.1 | 1.8 |
| N22 | 1.9 ± 0.34 | 2.7 ± 1.13 | 2.3 | 2.4 ± 0.5 | 1.8 ± 0.2 | 2.1 |
| Rasi | 2.2 ± 0.23 | 1.5 ± 0.44 | 1.9 | 2.2 ± 0 | 1.3 ± 0.04 | 1.8 |
| Sampada | 2.2 ± 0.63 | 2.7 ± 0.18 | 2.4 | 2.5 ± 0.3 | 2.1 ± 0.04 | 2.3 |
| Swarna | 2.5 ± 0.34 | 2.6 ± 0.07 | 2.6 | 3.1 ± 0.5 | 1.8 ± 0.1 | 2.4 |
| Vandana | 1.6 ± 0.55 | 2.1 ± 0.31 | 1.9 | 1.8 ± 0.5 | 1.2 ± 0.01 | 1.5 |
| Varadhan | 2.6 ± 0.33 | 2.3 ± 0.24 | 2.5 | 1.9 ± 0.2 | 0.9 ± 0.01 | 1.4 |
| **Mean** | 2.4 | 2 | 2.2 | 2.4 | 1.4 | 1.9 |
| HSD(Treat) | 0.35(P<0.05) | | | 0.12(P<0.01) | | |
| HSD(Variety) | 1.4(NS) | | | 0.4(P<0.01) | | |
| HSD(Tx V) | 2.31(NS) | | | 0.79(P<0.01) | | |
| CV(%) | 2.9 | | | 10.24 | | |

**Supplementary Table 2 : Effect of elevated temperature on (1) Chl a/b ratio and (2) Carotenoid content at vegetative stage and reproductive stage.**

| **(1)** | **Chl a/b** | | | | | |
| --- | --- | --- | --- | --- | --- | --- |
|  | Vegetative Stage | | | Reproductive Stage | | |
|  | Control | Elevated | Mean | Control | Elevated | Mean |
| BPT5204 | 4 ± 0.08 | 4.3 ± 0.77 | 4.2 | 3.2 ± 0.1 | 0.7 ± 0.3 | 2 |
| IR64 | 2.7 ± 0.4 | 2.6 ± 0.28 | 2.6 | 3.9 ± 1.4 | 1.6 ± 0.1 | 2.7 |
| Jaya | 3.4 ± 0.2 | 3.7 ± 0.05 | 3.6 | 4.7 ± 0.9 | 2.7 ± 0.4 | 3.7 |
| Krishnahamsa | 3.4 ± 0.32 | 3.4 ± 0.7 | 3.4 | 5.3 ± 0.5 | 3.2 ± 0.4 | 4.3 |
| MTU1010 | 3.7 ± 0.31 | 3.1 ± 0.13 | 3.4 | 6.1 ± 1.1 | 2 ± 0.3 | 4.1 |
| N22 | 4.1 ± 0.16 | 4.3 ± 0.52 | 4.2 | 1.8 ± 0.5 | 1.7 ± 0.1 | 1.7 |
| Rasi | 3.9 ± 0.24 | 4.3 ± 0.78 | 4.1 | 3.3 ± 0.1 | 3.7 ± 0.1 | 3.5 |
| Sampada | 3.1 ± 0.08 | 3.8 ± 0.49 | 3.5 | 4.3 ± 2.2 | 2.8 ± 0.1 | 3.5 |
| Swarna | 3.7 ± 0.22 | 1.8 ± 0.57 | 2.7 | 3.3 ± 0.1 | 2.1 ± 0.2 | 2.7 |
| Vandana | 3.3 ± 0.29 | 0.9 ± 0.03 | 2.1 | 1.7 ± 0.2 | 1.2 ± 0.3 | 1.5 |
| Varadhan | 2.1 ± 0.8 | 0.93 ± 0.1 | 1.5 | 6 ± 0.3 | 1.3 ± 0.3 | 3.6 |
| **Mean** | 3.4 | 3.0 | 3.2 | 4.0 | 2.1 | 3.0 |
| HSD(Treat) | 0.5(NS) | | | 0.08(P<0.01) | | |
| HSD(Variety) | 2.8(P<0.01) | | | 0.32(P<0.01) | | |
| HSD(T x V) | 3.6(NS) | | | 0.52(P<0.01) | | |
| CV(%) | 12.3 | | | 15.40 | | |

| **(2)** | **Carotenoids** | | | | | |
| --- | --- | --- | --- | --- | --- | --- |
|  | Vegetative Stage | | | Reproductive Stage | | |
|  | Control | Elevated | Mean | Control | Elevated | Mean |
| BPT5204 | 0.6 ± 0.04 | 0.3 ± 0.03 | 0.5 | 0.7 ± 0.1 | 0.5 ± 0.03 | 0.6 |
| IR64 | 0.5 ± 0.05 | 0.7 ± 0.15 | 0.6 | 0.6 ± 0.08 | 0.2 ± 0.09 | 0.4 |
| Jaya | 0.7 ± 0.02 | 0.6 ± 0.06 | 0.6 | 0.8 ± 0.18 | 0.1 ± 0.04 | 0.5 |
| Krishnahamsa | 0.8 ± 0.12 | 0.5 ± 0.11 | 0.6 | 1 ± 0.02 | 0.3 ± 0.04 | 0.6 |
| MTU1010 | 0.5 ± 0.01 | 0.3 ± 0.01 | 0.4 | 0.4 ± 0.38 | 0.5 ± 0.1 | 0.5 |
| N22 | 0.5 ± 0.06 | 0.7 ± 0.27 | 0.6 | 0.4 ± 0.22 | 0.2 ± 0.11 | 0.3 |
| Rasi | 0.6 ± 0.06 | 0.4 ± 0.09 | 0.5 | 0.8 ± 0.01 | 0.5 ± 0.02 | 0.7 |
| Sampada | 0.7 ± 0.29 | 0.6 ± 0.01 | 0.6 | 0.6 ± 0.07 | 0.5 ± 0.01 | 0.6 |
| Swarna | 0.6 ± 0.09 | 0.1 ± 0.51 | 0.4 | 0.7 ± 0.03 | 0.7 ± 0.02 | 0.7 |
| Vandana | 0.3 ± 0.22 | 0.4 ± 0.08 | 0.3 | 0.3 ± 0.01 | 0.05 ± 0.0 | 0.2 |
| Varadhan | 0.8 ± 0.31 | 0.4 ± 0.12 | 0.6 | 0.9 ± 0.05 | 0.3 ± 0.29 | 0.6 |
| **Mean** | 0.6 | 0.5 | 0.5 | 0.65 | 0.4 | 0.5 |
| HSD(Treat) | 0.15(P<0.05) | | | 0.42(P<0.01) | | |
| HSD(Variety) | 0.61(NS) | | | 1.69(P<0.01) | | |
| HSD(T x V) | 0.98(NS) | | | 2.72(P<0.01) | | |
| CV(%) | 5.7 | | | 22.2 | | |

**Supplementary Table 3: Effect of elevated temperature on CAT (catalase) activity** at maximum vegetative stage and reproductive stage.

|  | **CAT (µmol H_2_O_2_ oxidized min^-1^ g^-1^ protein)** | | | | | |
| --- | --- | --- | --- | --- | --- | --- |
|  | Vegetative Stage | | | Reproductive Stage | | |
|  | Control | Elevated | Mean | Control | Elevated | Mean |
| BPT5204 | 9.5 ± 0.8 | 14.9 ± 0.2 | 12.2 | 10.2 ± 1.5 | 8.6 ± 3.8 | 9.4 |
| IR64 | 12 ± 1.2 | 11.6 ± 2.4 | 11.8 | 9 ± 0.3 | 12.2 ± 2 | 10.6 |
| Jaya | 12.8 ± 1.7 | 11 ± 1.7 | 11.9 | 13.2 ± 0.3 | 8.7 ± 1.5 | 11 |
| Krishna hamsa | 9.9 ± 1.2 | 8.3 ± 2.3 | 9.1 | 6.8 ± 1.7 | 3.2 ± 0.5 | 5 |
| MTU1010 | 9.5 ± 0.4 | 7.1 ± 0.5 | 8.3 | 12.9 ± 1.8 | 6.9 ± 0.9 | 9.9 |
| N22 | 10.7 ± 1.9 | 11.4 ± 2.8 | 11 | 4.1 ± 1.1 | 8.9 ± 2 | 6.5 |
| Rasi | 11.6 ± 1.6 | 12.3 ± 0.3 | 11.9 | 22 ± 16.7 | 8.3 ± 4.0 | 15.2 |
| Sampada | 10.1 ± 1.4 | 13.8 ± 0.3 | 11.9 | 13.2 ± 0.6 | 6 ± 0.9 | 9.6 |
| Swarna | 10.7 ± 0.2 | 19.1 ± 1.2 | 14.9 | 12.2 ± 0.2 | 8.9 ± 0.8 | 10.5 |
| Vandana | 10.8 ± 2.4 | 3.8 ± 0.8 | 7.3 | 14.5 ± 2.8 | 10± 3.0 | 12.4 |
| Varadhan | 14.9 ± 1.7 | 9 ± 0.3 | 11.9 | 8 ± 0.5 | 22 ± 6 | 15 |
| **Mean** | 11.10 | 11.08 | 11.0 | 11.5 | 9.4 | 10.4 |
| HSD(Treat) | 1.93(NS) | | | 3.68(NS) | | |
| HSD(Variety) | 7.81(NS) | | | 14.91(P<0.05) | | |
| HSD(Treat x Variety) | 12.54(NS) | | | 23.94(P<0.01) | | |
| CV(%) | 5.7 | | | 5.05 | | |

**Supplementary Table 4: Effect of elevated temperature on (1) internal CO_2_ (Ci) (2) Ci/Ca at vegetative stage and reproductive stage.**

| **(1)** | **Internal CO_2_(Ci)** | | | | | |
| --- | --- | --- | --- | --- | --- | --- |
|  | Vegetative Stage | | | Reproductive Stage | | |
|  | Control | Elevated | Mean | Control | Elevated | Mean |
| BPT5204 | 311 ± 5 | 289 ± 8 | 300 | 270 ± 38 | 250 ± 18 | 260 |
| IR64 | 290 ± 25 | 248 ± 20 | 269 | 274 ± 57 | 326 ± 52 | 300 |
| Jaya | 293 ± 22 | 282 ± 22 | 288 | 315 ± 23 | 299 ± 11 | 307 |
| Krishnahamsa | 312 ± 17 | 342 ± 13 | 327 | 258 ± 14 | 301 ± 11 | 279 |
| MTU1010 | 272 ± 2 | 258 ± 5 | 265 | 318 ± 14 | 151 ± 15 | 235 |
| N22 | 260 ± 21 | 260 ± 7 | 260 | 307 ± 16 | 325 ± 22 | 316 |
| Rasi | 300 ± 22 | 254 ± 39 | 277 | 281 ± 23 | 312 ± 13 | 297 |
| Sampada | 316 ± 15 | 285 ± 4 | 301 | 288 ± 14 | 244 ± 22 | 266 |
| Swarna | 314 ± 10 | 248 ± 6 | 281 | 253 ± 32 | 301 ± 48 | 277 |
| Vandana | 161 ± 34 | 208 ± 51 | 185 | 305 ± 11 | 284 ± 19 | 295 |
| Varadhan | 303 ± 14 | 315 ± 6 | 309 | 275 ± 49 | 338 ± 26 | 307 |
| **Mean** | 285 | 272 | 278 | 286 | 285 | 285 |
| HSD(Treat) | 10.59(P<0.05) | | | 21.0(NS) | | |
| HSD(Variety) | 41.83 (P<0.01) | | | 84.1(P<0.05) | | |
| HSD(T x V) | 66.65 (NS) | | | 134(P<0.01) | | |
| CV(%) | 7.67 | | | 15 | | |

| **(2)** | **Ci/Ca** | | | | | |
| --- | --- | --- | --- | --- | --- | --- |
|  | Vegetative Stage | | | Reproductive Stage | | |
|  | Control | Elevated | Mean | Control | Elevated | Mean |
| BPT5204 | 0.8 ± 0.03 | 0.7 ± 0.01 | 0.8 | 0.72 ± 0.1 | 0.72 ± 0.07 | 0.72 |
| IR64 | 0.8 ± 0.06 | 0.6 ± 0.05 | 0.7 | 0.73 ± 0.15 | 0.88 ± 0.13 | 0.81 |
| Jaya | 0.7 ± 0.06 | 0.8 ± 0.06 | 0.8 | 0.84 ± 0.06 | 0.8 ± 0.03 | 0.82 |
| Krishna hamsa | 0.8 ± 0.04 | 0.9 ± 0.03 | 0.8 | 0.7 ± 0.04 | 0.81 ± 0.03 | 0.75 |
| MTU1010 | 0.7 ± 0.05 | 0.7 ± 0.01 | 0.8 | 0.85 ± 0.04 | 0.4 ± 0.4 | 0.63 |
| N22 | 0.7 ± 0.06 | 0.7 ± 0.01 | 0.7 | 0.79 ± 0.04 | 0.83 ± 0.05 | 0.81 |
| Rasi | 0.8 ± 0.05 | 0.7 ± 0.1 | 0.7 | 0.75 ± 0.06 | 0.83 ± 0.03 | 0.79 |
| Sampada | 0.8 ± 0.04 | 0.7 ± 0.01 | 0.8 | 0.77 ± 0.03 | 0.64 ± 0.05 | 0.71 |
| Swarna | 0.8 ± 0.03 | 0.7 ± 0.01 | 0.7 | 0.68 ± 0.08 | 0.79 ± 0.13 | 0.74 |
| Vandana | 0.4 ± 0.09 | 0.5 ± 0.13 | 0.5 | 0.79 ± 0.03 | 0.73 ± 0.06 | 0.76 |
| Varadhan | 0.8 ± 0.03 | 0.8 ± 0.02 | 0.8 | 0.74 ± 0.14 | 0.9 ± 0.07 | 0.82 |
| Mean | 0.7 | 0.7 | 0.7 | 0.76 | 0.76 | 0.76 |
| HSD(Treat) | 0.03(P<0.05) | | | 0.06(NS) | | |
| HSD(Variety) | 0.11(P<0.01) | | | 0.22(NS) | | |
| HSD(T x V) | 0.17(P<0.01) | | | 0.36(P<0.01) | | |
| CV(%) | 7.67 | | | 15 | | |

**Supplementary Table 5: Effect of elevated temperature on intrinsic water use efficiency (iWUE)** at vegetative stage and reproductive stage.

|  | **Intrinsic water use efficiency(** iWUE) | | | | | |
| --- | --- | --- | --- | --- | --- | --- |
|  | Vegetative Stage | | | Reproductive Stage | | |
|  | Control | Elevated | Mean | Control | Elevated | Mean |
| BPT5204 | 40 ± 3 | 37.2 ± 6 | 38.6 | 53 ± 22 | 63 ± 12 | 58 |
| IR64 | 47 ± 12 | 71.4 ± 12 | 59.2 | 34 ± 31 | 27 ± 11 | 31 |
| Jaya | 50.8 ± 13 | 41 ± 14 | 45.9 | 27 ± 14 | 37 ± 6 | 32 |
| Krishna hamsa | 38.7 ± 10 | 22.3 ± 8 | 30.5 | 58 ± 9 | 36 ± 6 | 47 |
| MTU1010 | 57.8 ± 10 | 67.7 ± 4 | 62.7 | 35 ± 9 | 64 ± 85 | 49 |
| N22 | 67.2 ± 13 | 73.4 ± 4 | 70.3 | 52 ± 9 | 29 ± 12 | 41 |
| Rasi | 46 ± 12 | 68.8 ± 25 | 57.4 | 73 ± 11 | 28 ± 7 | 50 |
| Sampada | 35.5 ± 10 | 50.4 ± 2 | 42.9 | 46 ± 7 | 71 ± 12 | 58 |
| Swarna | 55 ± 6 | 42.2 ± 3 | 48.6 | 64 ± 18 | 27 ± 32 | 46 |
| Vandana | 122 ± 23 | 93 ± 33 | 108 | 42 ± 6 | 50 ± 15 | 46 |
| Varadhan | 48.2 ± 8 | 36.4 ± 3 | 42.3 | 41 ± 29 | 34 ± 12 | 38 |
| **Mean** | 55.3 | 54.9 | 55.1 | 48 | 42 | 45 |
| HSD(Treat) | 6.46(NS) | | | 11.81(NS) | | |
| HSD(Variety) | 25.49(P<0.01) | | | 46.62(NS) | | |
| HSD(TxV) | 40.62(P<0.05) | | | 74.28(P<0.01) | | |

**Supplementary Table 6: Effect of elevated temperature on (1) intrinsic efficiency of PhotosystemII (Fv'/Fm') at maximum vegetative stage and reproductive stage.**

| **(1)** | **Intrinsic efficiency of PhotosystemII Fv'/Fm'** | | | | | |
| --- | --- | --- | --- | --- | --- | --- |
|  | Vegetative Stage | | | Reproductive Stage | | |
|  | Control | Elevated | Mean | Control | Elevated | Mean |
| BPT5204 | 0.44 ± 0.01 | 0.38 ± 0.04 | 0.47 | 0.45 ± 0.02 | 0.38 ± 0.02 | 0.42 |
| IR64 | 0.46 ± 0.04 | 0.44 ± 0.05 | 0.46 | 0.56 ± 0.01 | 0.54 ± 0.05 | 0.57 |
| Jaya | 0.52 ± 0.03 | 0.45 ± 0.01 | 0.48 | 0.77 ± 0.2 | 0.46 ± 0.01 | 0.61 |
| Krishna hamsa | 0.55 ± 0.01 | 0.44 ± 0.05 | 0.41 | 0.52 ± 0.01 | 0.45 ± 0.02 | 0.48 |
| MTU1010 | 0.51 ± 0.03 | 0.49 ± 0.07 | 0.55 | 0.51 ± 0.07 | 0.46 ± 0.06 | 0.48 |
| N22 | 0.61 ± 0.03 | 0.60 ± 0.04 | 0.64 | 0.48 ± 0.04 | 0.33 ± 0.03 | 0.4 |
| Rasi | 0.56 ± 0.04 | 0.46 ± 0.12 | 0.52 | 0.5 ± 0.03 | 0.45 ± 0.05 | 0.48 |
| Sampada | 0.69 ± 0.07 | 0.47 ± 0.09 | 0.51 | 0.48 ± 0.01 | 0.49 ± 0.03 | 0.48 |
| Swarna | 0.51 ± 0.03 | 0.43 ± 0.05 | 0.47 | 0.53 ± 0.05 | 0.43 ± 0.06 | 0.48 |
| Vandana | 0.61 ± 0.05 | 0.52 ± 0.04 | 0.48 | 0.47 ± 0.03 | 0.4 ± 0.04 | 0.43 |
| Varadhan | 0.47 ± 0.01 | 0.43 ± 0.02 | 0.45 | 0.576 ± 0.08 | 0.55 ± 0.03 | 0.56 |
| **Mean** | 0.54 | 0.46 | 0.5 | 0.53 | 0.46 | 0.49 |
| HSD(Treat) | 0.02(NS) | | | 0.028(P<0.01) | | |
| HSD(Variety) | 0.1(P<0.01) | | | 0.11(P<0.01) | | |
| HSD(Tx V | 0.16(P<0.01) | | | 0.18(P<0.01) | | |
| CV(%) | 9.94 | | | 11.78 | | |

**Supplementary Table 7: Effect of elevated temperature on (1) In vivo quantum yield of PSII photochemistry (*Φ*_PSII_) (2) Quantum yield of CO2 assimilation (*Φ*Co_2_) (3) Coefficient of Photochemical quenching *(qP)* and (4) Coefficient of Non-photochemical quenching (*qN)*** at vegetative stage and reproductive stage.

| **(1)** | **In vivo quantum yield of PSII photochemistry (ΦPS_II_)** | | | | | |
| --- | --- | --- | --- | --- | --- | --- |
|  | **Vegetative Stage** | | | **Reproductive Stage** | | |
|  | Control | Elevated | Mean | Control | Elevated | Mean |
| BPT5204 | 0.16 ± 0.04 | 0.14 ± 0.04 | 0.26 | 0.17 ± 0.03 | 0.15 ± 0.04 | 0.16 |
| IR64 | 0.26 ± 0.03 | 0.17 ± 0.1 | 0.22 | 0.26 ± 0 | 0.25 ± 0.02 | 0.26 |
| Jaya | 0.23 ± 0.02 | 0.22 ± 0.04 | 0.18 | 0.27 ± 0.01 | 0.23 ± 0.01 | 0.25 |
| Krishna hamsa | 0.27 ± 0.02 | 0.24 ± 0.02 | 0.25 | 0.32 ± 0.01 | 0.25 ± 0.04 | 0.29 |
| MTU1010 | 0.32 ± 0.01 | 0.25 ± 0.01 | 0.27 | 0.21 ± 0.01 | 0.18 ± 0.07 | 0.19 |
| N22 | 0.30 ± 0.04 | 0.26 ± 0.04 | 0.3 | 0.32 ± 0.04 | 0.19 ± 0.03 | 0.25 |
| Rasi | 0.28 ± 0.02 | 0.23 ± 0.04 | 0.25 | 0.3 ± 0.03 | 0.18 ± 0.02 | 0.24 |
| Sampada | 0.26 ± 0.05 | 0.23 ± 0.07 | 0.22 | 0.2 ± 0.03 | 0.14 ± 0.03 | 0.17 |
| Swarna | 0.24 ± 0.04 | 0.17 ± 0.06 | 0.25 | 0.24 ± 0.02 | 0.17 ± 0.01 | 0.2 |
| Vandana | 0.35 ± 0.04 | 0.33 ± 0.05 | 0.33 | 0.33 ± 0.04 | 0.26 ± 0.04 | 0.29 |
| Varadhan | 0.25 ± 0.03 | 0.24 ± 0.05 | 0.24 | 0.21 ± 0.03 | 0.23 ± 0.03 | 0.22 |
| **Mean** | 0.27 | 0.23 | 0.25 | 0.25 | 0.2 | 0.23 |
| HSD(Treat) | 0.02P<0.01) | | | 0.015504(P<0.01) | | |
| HSD(Variety) | 0.09(P<0.01) | | | 0.061216(P<0.01) | | |
| HSD(Tx V) | 0.14(NS) | | | 0.09754(P<0.01) | | |
| CV(%) | 17.38067 | | | 13.64 | | |

| **(2)** | **Quantum yield of CO2 assimilation (Φ_Co2_)** | | | | | |
| --- | --- | --- | --- | --- | --- | --- |
|  | **Vegetative Stage** | | | **Reproductive Stage** | | |
|  | Control | Elevated | Mean | Control | Elevated | Mean |
| BPT5204 | 0.005 | 0.005 | 0.0051 | 0.005 | 0.004 | 0.005 |
| IR64 | 0.005 | 0.003 | 0.004 | 0.018 | 0.010 | 0.01 |
| Jaya | 0.009 | 0 .007 | 0 .008 | 0.013 | 0.008 | 0.011 |
| Krishna hamsa | 0.011 | 0.01 | 0.011 | 0.014 | 0.011 | 0.012 |
| MTU1010 | 0.014 | 0.005 | 0.01 | 0.01 | 0.008 | 0.009 |
| N22 | 0.004 | 0.0031 | 0.003 | 0.017 | 0.006 | 0.012 |
| Rasi | 0.011 | 0.004 | 0.007 | 0.015 | 0.011 | 0.013 |
| Sampada | 0.013 | 0.005 | 0.009 | 0.007 | 0.008 | 0.007 |
| Swarna | 0.0061 | 0.006 | 0.006 | 0.009 | 0.006 | 0.008 |
| Vandana | 0.008 | 0.006 | 0.007 | 0.016 | 0.01 | 0.013 |
| Varadhan | 0.012 | 0.011 ± | 0.012 | 0.01 | 0.012 | 0.011 |
| **Mean** | 0.009 | 0.006 | 0.007 | 0.011 | 0.009 | 0.010 |
| HSD(Treat) | 0.001(P<0.01) | | | 0.001689(P<0.01) | | |
| HSD(Variety) | 0.006(P<0.01) | | | 0.00667(P<0.01) | | |
| HSD(T x V) | 0.01(P<0.05) | | | 0.010628(P<0.05) | | |
| CV(%) | 17.59 | | | 14.05 | | |

| **(3)** | **Coefficient of Photochemical quenching (*qP*)** | | | | | |
| --- | --- | --- | --- | --- | --- | --- |
|  | Vegetative Stage | | | Reproductive Stage | | |
|  | Control | Elevated | Mean | Control | Elevated | Mean |
| BPT5204 | 0.37 ± 0.09 | 0.37 ± 0.04 | 0.37 | 0.43 ± 0.06 | 0.34 ± 0.13 | 0.39 |
| IR64 | 0.58 ± 0.12 | 0.39 ± 0.15 | 0.48 | 0.48 ± 0.01 | 0.42 ± 0.07 | 0.45 |
| Jaya | 0.48 ± 0.03 | 0.49 ± 0.07 | 0.49 | 0.50 ± 0.07 | 0.36 ± 0 | 0.43 |
| Krishna hamsa | 0.51 ± 0.05 | 0.51 ± 0.02 | 0.51 | 0.63 ± 0.04 | 0.56 ± 0.1 | 0.59 |
| MTU1010 | 0.62 ± 0.03 | 0.48 ± 0.08 | 0.55 | 0.42 ± 0.07 | 0.38 ± 0.1 | 0.4 |
| N22 | 0.68 ± 0.09 | 0.39 ± 0.04 | 0.53 | 0.67 ± 0.04 | 0.55 ± 0.04 | 0.61 |
| Rasi | 0.50 ± 0.07 | 0.45 ± 0.06 | 0.47 | 0.6 ± 0.07 | 0.4 ± 0.04 | 0.5 |
| Sampada | 0.38 ± 0.04 | 0.37 ± 0.11 | 0.37 | 0.42 ± 0.07 | 0.29 ± 0.08 | 0.35 |
| Swarna | 0.46 ± 0.05 | 0.39 ± 0.14 | 0.43 | 0.45 ± 0.04 | 0.4 ± 0.06 | 0.43 |
| Vandana | 0.59 ± 0.01 | 0.60 ± 0.11 | 0.60 | 0.69 ± 0.04 | 0.65 ± 0.02 | 0.67 |
| Varadhan | 0.38 ± 0.06 | 0.38 ± 0.1 | 0.38 | 0.38 ± 0.01 | 0.39 ± 0.07 | 0.38 |
| **Mean** | 0.55 | 0.47 | 0.47 | 0.49 | 0.45 | 0.47 |
| HSD(Treat) | 0.04(P<0.01) | | | 0.03(P<0.01) | | |
| HSD(Variety) | 0.16(P<0.01) | | | 0.12(P<0.01) | | |
| HSD(T x V) | 0.25(P<0.1) | | | 0.197(P<0.01) | | |
| CV(%) | 15.76 | | | 13.38 | | |

| **(4)** | **Coefficient of Non-photochemical quenching (** *qN)* | | | | | |
| --- | --- | --- | --- | --- | --- | --- |
|  | Vegetative Stage | | | Reproductive Stage | | |
|  | Control | Elevated | Mean | Control | Elevated | Mean |
| BPT5204 | 0.769 ± 0.07 | 0.766 ± 0.08 | 0.768 | 0.76 ± 0.03 | 0.75 ± 0.03 | 0.76 |
| IR64 | 0.67 ± 0.06 | 0.63 ± 0.05 | 0.65 | 0.46 ± 0.08 | 0.53 ± 0.1 | 0.5 |
| Jaya | 0.58 ± 0.04 | 0.70 ± 0.02 | 0.64 | 0.29 ± 0.07 | 0.71 ± 0.02 | 0.5 |
| Krishna hamsa | 0.54 ± 0.02 | 0.71 ± 0.08 | 0.62 | 0.55 ± 0.05 | 0.71 ± 0.02 | 0.63 |
| MTU1010 | 0.55 ± 0.07 | 0.59 ± 0.14 | 0.57 | 0.64 ± 0.11 | 0.68 ± 0.11 | 0.66 |
| N22 | 0.381 ± 0.11 | 0.67 ± 0.17 | 0.52 | 0.71 ± 0.07 | 0.86 ± 0.02 | 0.79 |
| Rasi | 0.45 ± 0.08 | 0.67 ± 0.12 | 0.56 | 0.62 ± 0.02 | 0.74 ± 0.04 | 0.68 |
| Sampada | 0.29 ± 0.14 | 0.58 ± 0.17 | 0.43 | 0.71 ± 0.03 | 0.73 ± 0.01 | 0.72 |
| Swarna | 0.60 ± 0.06 | 0.74 ± 0.05 | 0.67 | 0.59 ± 0.07 | 0.74 ± 0.06 | 0.66 |
| Vandana | 0.30 ± 0.08 | 0.45 ± 0.12 | 0.38 | 0.66 ± 0.06 | 0.82 ± 0.05 | 0.74 |
| Varadhan | 0.44 ± 0.03 | 0.44 ± 0.02 | 0.44 | 0.58 ± 0.15 | 0.57 ± 0.05 | 0.57 |
| **Mean** | 0.61 | 0.50 | 0.57 | 0.6 | 0.69 | 0.65 |
| HSD(Treat) | 0.05(P<0.01) | | | 0.03(P<0.01) | | |
| HSD(Variety) | 0.18(P<0.01) | | | 0.12(P<0.01) | | |
| HSD(Treat x Variety) | 0.29(P<0.01) | | | 0.20(P<0.01) | | |
| CV(%) | 15.5 | | | 10.17 | | |

**Supplementary Table 8**: Effect of elevated temperature on plant height, tiller number and panicle number.

|  | **Plant height (cm)** | | | **Tiller/Panicle number per hill** | | |
| --- | --- | --- | --- | --- | --- | --- |
|  | Control | ETS | Mean | Control | ETS | Mean |
| BPT5204 | 73 ± 6 | 70 ± 9 | 72 | 11 ± 2 | 9.3 ± 3 | 10.2 |
| IR64 | 78 ± 10 | 80 ± 2 | 79 | 16 ± 1 | 15.4 ± 3 | 15.7 |
| Jaya | 77 ± 4 | 75 ± 8 | 76 | 12.4 ± 1 | 9.7 ± 1 | 12.4 |
| Krishna hamsa | 74 ± 5 | 77 ± 10 | 76 | 16.9 ± 4 | 19.8 ± 2 | 18.4 |
| MTU1010 | 84 ± 6 | 82 ± 8 | 83 | 10 ± 1 | 11 ± 0 | 10.5 |
| N22 | 75 ± 0 | 90 ± 1 | 83 | 17 ± 2 | 18.7 ± 3 | 17.8 |
| Rasi | 84 ± 6 | 85 ± 6 | 85 | 11 ± 3 | 13.3 ± 2 | 12.2 |
| Sampada | 79 ± 4 | 82 ± 3 | 81 | 14.3 ± 3 | 13.7 ± 1 | 14 |
| Swarna | 82 ± 4 | 90 ± 2 | 86 | 15 ± 1 | 14.7 ± 3 | 14.8 |
| Vandana | 86 ± 6 | 88 ± 5 | 87 | 18 ± 4 | 18.3 ± 2 | 18.2 |
| Varadhan | 92 ± 2 | 85 ± 6 | 89 | 10.3 ± 1 | 12 ± 1 | 11.2 |
| **Mean** | 80 | 82 | 81 | 13.8 | 14.2 | 14.1 |
| HSD(Treat) | 2.8(NS) | | | 1.0(P<0.05) | | |
| HSD(Variety) | 11.4P<0.01) | | | 3.99P<0.01) | | |
| HSD(T x V) | 18.2P<0.05) | | | 6.3P<0.01) | | |
| CV(%) | 7.2 | | | 14.5 | | |

**Supplementary Table 9: Correlation analysis performed between grain yield recorded and different physiological parameters studied at reproductive stage under elevated temperature.**

|  | *Grain yield/hill* | *Spikelet fertility* | *RI* | *SOD* | *CAT* | *POD* | *Chlorophyll* | P_N_ | *Gs* | *E* | *Fv/fm* | *fv'/fm'* | Φ_PSII_ | Φ_CO2_ | *ETR* |
| --- | --- | --- | --- | --- | --- | --- | --- | --- | --- | --- | --- | --- | --- | --- | --- |
| Grain yield | 1.00 |  |  |  |  |  |  |  |  |  |  |  |  |  |  |
| Spikelet fertility | 0.67 | 1.00 |  |  |  |  |  |  |  |  |  |  |  |  |  |
| RI | -0.56 | -0.35 | 1.00 |  |  |  |  |  |  |  |  |  |  |  |  |
| SOD | -0.24 | -0.28 | 0.54 | 1.00 |  |  |  |  |  |  |  |  |  |  |  |
| CAT | 0.24 | 0.07 | -0.49 | 0.19 | 1.00 |  |  |  |  |  |  |  |  |  |  |
| POD | -0.23 | -0.09 | 0.40 | 0.02 | -0.56 | 1.00 |  |  |  |  |  |  |  |  |  |
| Chlorophyll | -0.22 | -0.41 | 0.13 | -0.30 | -0.21 | 0.38 | 1.00 |  |  |  |  |  |  |  |  |
| P_N_ | 0.23 | 0.44 | 0.10 | -0.01 | -0.19 | -0.13 | -0.64 | 1.00 |  |  |  |  |  |  |  |
| Gs | 0.19 | 0.65 | 0.15 | -0.09 | 0.03 | -0.11 | -0.38 | 0.76 | 1.00 |  |  |  |  |  |  |
| E | 0.46 | 0.66 | -0.28 | -0.16 | 0.44 | -0.30 | -0.29 | 0.51 | 0.79 | 1.00 |  |  |  |  |  |
| Fv/fm | -0.43 | -0.37 | 0.02 | 0.09 | 0.37 | -0.61 | 0.17 | -0.28 | -0.10 | -0.12 | 1.00 |  |  |  |  |
| fv'/fm' | 0.32 | 0.15 | -0.63 | -0.41 | 0.61 | -0.61 | -0.02 | 0.20 | 0.29 | 0.66 | 0.31 | 1.00 |  |  |  |
| Φ_PSII_ | 0.18 | 0.34 | -0.16 | 0.02 | 0.35 | -0.27 | -0.74 | 0.67 | 0.59 | 0.60 | -0.26 | 0.39 | 1.00 |  |  |
| Φ_CO2_ | 0.30 | 0.36 | -0.34 | -0.15 | 0.41 | -0.27 | -0.37 | 0.54 | 0.58 | 0.86 | -0.19 | 0.71 | 0.73 | 1.00 |  |
| ETR | 0.26 | 0.38 | -0.16 | 0.04 | 0.33 | -0.30 | -0.79 | 0.73 | 0.61 | 0.59 | -0.26 | 0.36 | 0.99 | 0.69 | 1.00 |
