# Supplementary material for: Integrated Physiological, Biochemical, and Molecular Analysis Identifies Important Traits and Mechanisms Associated with Differential Response of Rice Genotypes to Elevated Temperature
Source: Front Plant Sci. 2015 Nov 27;6:1044. doi: 10.3389/fpls.2015.01044 (PMC4661239; doi:10.3389/fpls.2015.01044)
Supplement: Supplementary file 3 [file DataSheet1.DOC]

**Membrane thermo stability (MTS)**

The MTS of leaf samples was measured by the method described by Haque et al. 2009. The leaf samples of control and elevated temperature treatments were washed three times and collected in 15ml sterile conical tubes with 10 ml de-ionized water. Two sets of each sample were prepared, one set designated as control was maintained at 28°C while other set was treated in water bath at 52°C for one hour. Here, three replications were maintained for both the sets. After the treatment, control and treated tubes were kept at room temperature for 24h. The initial conductance was measured using conductivity meter. Thereafter, all the tubes were autoclaved at 121° C at 15 lbs for 20 min and the next day final conductance was measured. This ensures complete electrolyte leakage from the plant tissue. The relative injury (RI) was calculated using the following formula:

Injury (%) = E-I/F x 100;

Where, I = initial conductance; E= elevated temperature conductance; F= final conductance after autoclaving

**Chlorophyll and carotenoids estimation**

Photosynthesis pigments were estimated by method described by Lichtenthaler and Wellburn (1983). Chlorophyll was extracted from 100mg of leaf sample in 25ml of 80% acetone. The absorbance of *Chl a, Chl b* and carotenoid content was measured using UV-Spectrophotometer at 663.2, 646.8 and 470 nm wavelengths, respectively.

**Enzymes assays**

For antioxidant enzymes assays, leaf samples of eleven cultivars were collected in vegetative (40d after transplantation) and reproductive (5d after anthesis) phases from control and elevated temperature samples. One gram of fresh leaf tissue was homogenized in 0.1 M phosphate buffer (pH 7.5) containing 0.5 mM EDTA in pre chilled mortar and pestle. The homogenized tissue was centrifuged at 16,000 g for 30 min at 4°C and the collected supernatant was used for enzyme assays.

SOD (super oxide dismutase) activity assay

SOD activity was assayed by following the method given by Dhindsa et al. (1981). 3.0 ml of reaction mixture consists of 50mM phosphate buffer (pH 7.8) containing 0.1 mM EDTA, 50mM sodium carbonate, 75uM of NBT and 13.3mM of methionine. The absorbance of blue coloured formazone formed by nitro-blue tetrazolium chloride (NBT) and superoxide radical (O_2_^-.^) was measured in UV-visible spectrophotometer at 560 nm. One unit of SOD activity was expressed as the amount of enzyme required to inhibit the absorbance of NBT by 50%. The activity was expressed as unit min^-1^ g FW^-1^.

CAT (catalase) activity assay

CAT activity was measured by the method described by Aebi (1984). 3.0ml of assay mixture consists of 100mM phosphate buffer (pH7.0), 12.5mM of H_2_O_2_ and 50ul of enzyme extract and the rate of H_2_O_2_ decomposition was measured at 240 nm in UV spectrophotometer. It was measured every one minute for four minutes and decreased absorbance was recorded over a time period and it was expressed in µmol H_2_O_2_ oxidized min^-1^g FW^-1^.

POD (peroxidase) activity assay

POD activity was assayed according to the method Castillo et al. (1984), where 3.0ml of reaction mixture consists of 50mM phosphate buffer (pH 6.1), 16mM guiacol, 2mM H_2_O_2_ and 100ul of enzyme extract. The increased absorbance due to oxidation of guaiacol to tetraguaiacol was measured at 470 nm at every one minute for four minutes and the activity was expressed as unit min^-1^ g FW^-1^.

**Gaseous exchange parameters**

Leaf photosynthetic parameters were measured on fully matured leaves at vegetative and reproductive phases using LI6400XT portable photosynthesis measuring system (LI-COR Environmental,USA) connected to Leaf Chamber Fluorometer (6400-40, LI-COR, USA), which was used as a light source. Leaf temperature was maintained at 35°C, and PAR (photosynthetically active radiation) was maintained at 1,000 μmol m–^2^s–^1^. Measurements were made at ambient CO_2_ levels. The mean CO_2_ concentration during measurements was 387 μmol mol^–1^.

**Days to 50% flowering and days to maturity**

The number of days taken for 50% of plants to flower in each cultivar was noted as days to 50% flowering (both at control and elevated temperature) and was expressed in days. The number of days taken from sowing to physiological maturity was expressed as days to maturity.

**Plant height, number of panicles**

Plant height (cm) was measured using long scale. Three hills of each variety were selected from both control and treated. The panicles were separated from the plants for counting.

**Filled grains/panicle and spikelet sterility (%)**

The filled grains were separated from the panicle and were counted using seed counter and were expressed as number of filled grains panicle^-1^ and spikelet sterility (%) was calculated using the formulae.

Number of unfilled grains / Total number of spikelets×100.

**1000 grain weight and grain yield/plant (g pl^-1^)**

1000 grain weight was recorded by weighing thousand grains and expressed in g. For grain yield, panicles from treatment and control plants were harvested, sun dried, threshed, cleaned and weight of grains was recorded and expressed in g pl^-1^.

1. Aebi H (1984) Catalase in vitro. Methods in Enzy 105: 121-126
2. Castillo FI. Penel I, Greppin H (1984) Peroxidase release induced by ozone in Sedum album leaves. Plant Physiol 74: 846-851
3. Dhindsa RA, Plumb-Dhindsa P, Thorpe TA (1981) Leaf senescence: Correlated with increased permeability and lipid peroxidation, and decreased levels of superoxide dismutase and catalase. J Exp Bot 126: 93-101
4. Haque M, Hasan M, Rajib M, Hasan M (2009) Identification of cultivable heat tolerant wheat genotypes suitable for Patuakhali district in Bangladesh. J Bangladesh Agril Univ 7(2): 241–246
5. Lichtenthaler HK, Wellburn AR (1983) Determination of carotenoids and chlorophyll a and b of leaf extracts in different solvents. Biochem Soc Trans 11: 591-592
